# Quercetin Ameliorates Testicular Damage in Zucker Diabetic Fatty Rats through Its Antioxidant, Anti-Inflammatory and Anti-Apoptotic Properties

**DOI:** 10.3390/ijms232416056

**Published:** 2022-12-16

**Authors:** Eva Tvrdá, Ján Kováč, Kristína Ferenczyová, Barbora Kaločayová, Michal Ďuračka, Filip Benko, Viera Almášiová, Monika Barteková

**Affiliations:** 1 Institute of Biotechnology, Faculty of Biotechnology and Food Sciences, Slovak University of Agriculture in Nitra, Tr. A. Hlinku 2, 949 76 Nitra, Slovakia; 2 Institute of Applied Biology, Faculty of Biotechnology and Food Sciences, Slovak University of Agriculture in Nitra, Tr. A. Hlinku 2, 949 76 Nitra, Slovakia; 3 Institute for Heart Research, Centre of Experimental Medicine, Slovak Academy of Sciences, Dúbravská cesta 9, 841 04 Bratislava, Slovakia; 4 AgroBioTech Research Centre, Slovak University of Agriculture in Nitra, Tr. A. Hlinku 2, 949 76 Nitra, Slovakia; 5 Department of Morphological Disciplines, University of Veterinary Medicine and Pharmacy in Košice, Komenského 73, 041 81 Košice, Slovakia

**Keywords:** Zucker diabetic fatty rats, quercetin, testes, oxidative stress, inflammation, apoptosis, diabetes mellitus

## Abstract

The aim of this study was to investigate the effects of quercetin (QUE) on the testicular architecture as well as markers of oxidative, inflammatory, and apoptotic profile of male gonads in Zucker diabetic fatty (ZDF) rats suffering from Type 2 diabetes mellitus in the absence or presence of obesity. QUE was administered orally at a dose of 20 mg/kg/day for 6 weeks. Morphometric analysis revealed that QUE treatment led to an improvement in testicular appearance, particularly in the case of Obese ZDF rats. Furthermore, a significant stabilization of the antioxidant capacity (*p* < 0.05), superoxide dismutase and catalase activity (*p* < 0.01), with a concomitant decrease in lipid peroxidation (*p* < 0.05) were observed in Obese ZDF animals exposed to QUE. Our data also indicate a significant decline in the levels of interleukin (IL)-1 (*p* < 0.05), IL-6 (*p* < 0.01) and tumor necrosis factor alpha (*p* < 0.001) following QUE supplementation to Obese ZDF rats in comparison with their respective control. Finally, a significant down-regulation of the pro-apoptotic BAX protein (*p* < 0.0001) was observed in Obese ZDF rats administered with QUE, while a significant Bcl-2 protein overexpression (*p* < 0.0001) was recorded in Lean ZDF animals when compared to their untreated control. As such, our results suggest that QUE is a potentially beneficial agent to reduce testicular damage in ZDF rats with Type 2 diabetes mellitus by decreasing oxidative stress, chronic inflammation, and excessive cell loss through apoptosis.

## 1. Introduction

Type 2 diabetes mellitus (DM) may be defined as a cluster of metabolic ailments arising from abnormalities in the metabolism of carbohydrates and/or lipids which result in a deficient insulin secretion and subsequent insulin resistance [1,2,3]. Within this condition target cells become unable to absorb glucose, leading to the occurrence of hyperglycemia [3]. Type 2 DM currently accounts for more than 90% of all DM cases, and it is regarded as the most prevalent global metabolic disease, with an estimated 537 million adults being affected worldwide [4]. The etiology of the ailment is still not fully elucidated; however, a solid body of evidence indicates that obesity contributes significantly to the risk for the onset of Type 2 DM. Indeed, a recent review by Reed et al. [3] suggests that obese individuals are up to 80 times more likely to develop Type 2 DM in comparison to those with a normal body mass index. Metabolic syndrome is a frequently observed phenomenon interlinking Type 2 DM with obesity, referring to an array of pathophysiologies including hyperinsulinemia, hyperglycemia, insulin resistance, hypertension, and hypercholesterolemia [5]. On a cellular and molecular level, persistently elevated concentrations of blood sugar may accelerate the accumulation of advanced glycated end products and polyols, reactive oxygen species (ROS) generation, chronic inflammation, and cell death through apoptosis or necrosis [3,5].

A frequently reported consequence of Type 2 DM as well as obesity is a decreased ability in males to reproduce [6,7]. Functionally, male gonads are characterized by a continuous process of sperm production with high energy demands and a remarkable metabolic activity of male germ cells, which on the other hand renders them highly vulnerable to any external or internal disturbance of the physiological milieu [6]. A recent meta-analysis [8] has unraveled that both obesity and diabetes are associated with a reduced semen volume, sperm count and concentration, and alterations to the sperm motility and decreased testosterone levels, all of which are indicative of testicular dysfunction and spermatogenic disruption. The underlying mechanisms of such pathophysiology have been previously linked to endocrinopathy, aromatization activity, hypoxia, thermal stress, and increased angiogenesis in the testes, as well as inflammatory and obstructive elements of testicular and epididymal architecture [6,7].

Various strategies ranging from conventional drugs to natural products and dietary supplements have been studied in the prevention and/or management of infertility, among which flavonoids have received a renewed interest because of a wide array of their beneficial effects on human health [9]. In particular, quercetin (3,3′,4′,5,7-pentahydroxyflavone; QUE), a flavonoid found in a variety of commonly consumed fruits and vegetables, is one of the most powerful ROS scavengers [10], presenting with strong antioxidant and cytoprotective effects in different cellular models [11], including male reproductive cells [12,13]. Additionally, the molecule exhibits anti-peroxidative, anti-inflammatory, anti-ischemic, and anti-apoptotic effects [14] in addition to its potential in the prevention of β-cell damage and reduction of blood glucose in diabetic animals [15,16]. 

Evidence collected from previous in vivo studies indicates that QUE administration to diabetic males led to reduced testicular damage by decreasing oxidative stress and preventing excessive cell death [17,18,19]. Nevertheless, all currently available and relevant reports have been based on rats subjected to streptozotocin (STZ) that causes pancreatic β-cell destruction and is widely used to mimic symptoms consistent with Type 1 DM [20]. Conversely, animal models selected for our study were the Zucker diabetic fatty (ZDF) rats carrying a mutation in the leptin receptor gene, which causes leptin to become unable to bind to the ventromedial nuclei in the brain. Consequently, the animals will suffer from a consistent hunger and develop obesity when fed, which will eventually be followed by the onset of Type 2 DM. As such, ZDF rats are considered to be a suitable model to study the pathophysiology of Type 2 DM and the effects of potential therapeutic strategies for the prevention or management of the disease [21,22].

This study aimed to investigate the effects of QUE supplementation on the architecture and biochemical profile of testicular tissue collected from ZDF rats, with a special emphasis on selected oxidative, apoptotic, and inflammatory markers which have been suggested to be involved in male subfertility associated with Type 2 DM.

## 2. Results

### 2.1. Histology and Morphometry

**Figure 1:**
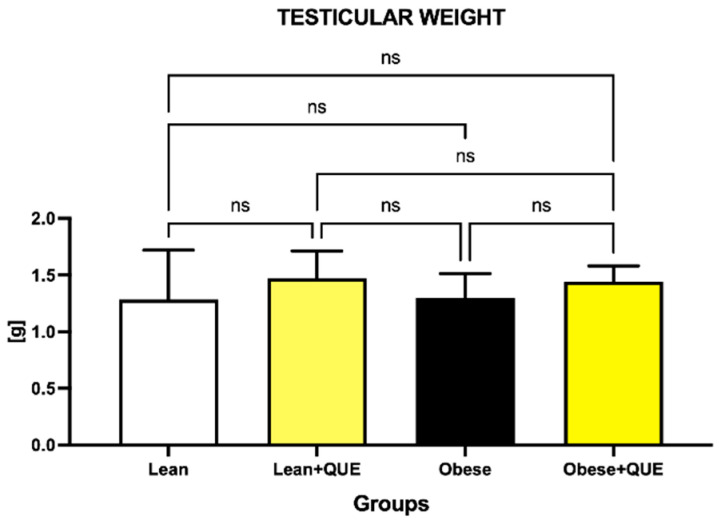
Testicular weight of the control and experimental groups. Data are presented as mean ± standard deviation. Lean–ZDF lean controls, Lean + QUE–ZDF lean rats supplemented with QUE, Obese–ZDF obese controls, Obese + QUE–ZDF obese rats supplemented with QUE. ns—non-significant.

Data depicting the testicular weight are shown in Figure 1. A lower testicular weight was observed in the Obese control in comparison to the Lean control; however, no significant differences were detected. QUE administration led to an increase in the weight of male gonads in both experimental groups when compared to their respective controls; nevertheless, these changes were non-significant.

**Figure 2:**
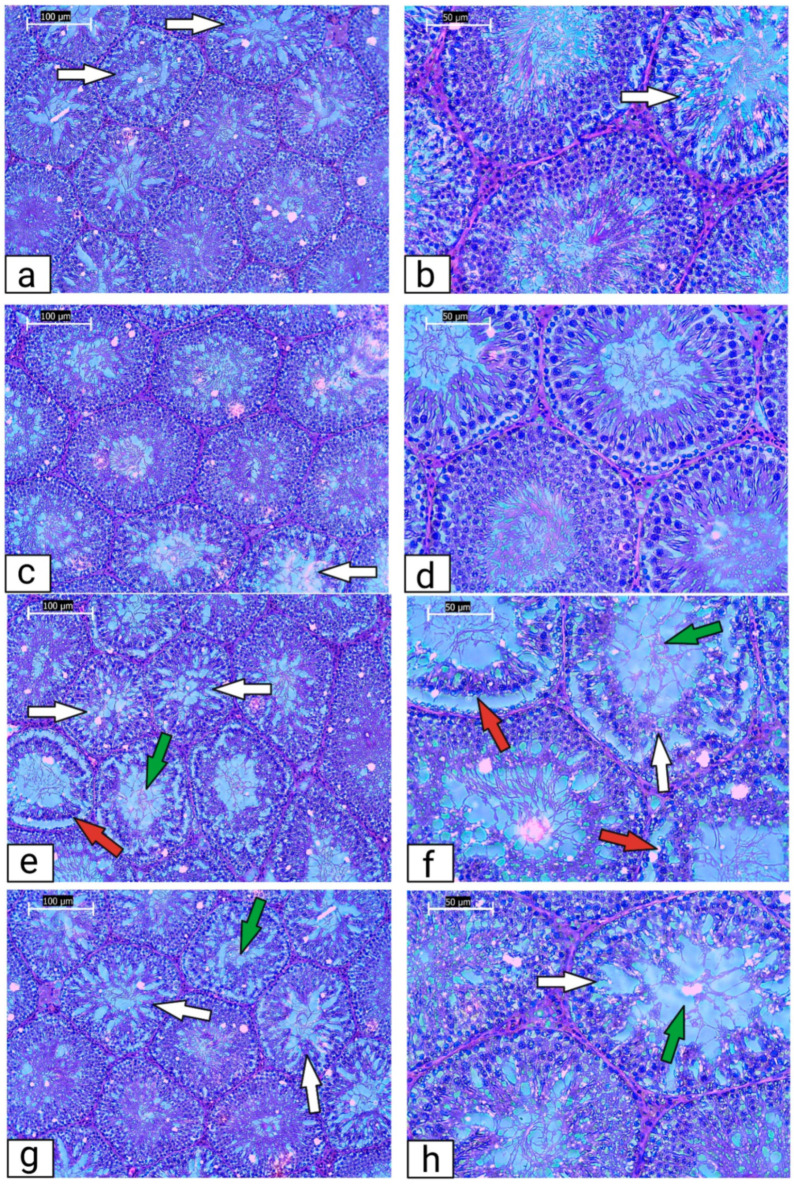
Representative photomicrographs of testicular tissue collected from (**a**,**b**) ZDF Lean control (*n* = 8); (**c**,**d**) ZDF Lean rats supplemented with QUE (*n* = 9); (**e**,**f**) ZDF Obese control (*n* = 13); and (**g**,**h**) ZDF Obese rats supplemented with QUE (*n* = 14). Several seminiferous tubules in the Lean control and Lean + QUE group showed signs of disruption in the seminal epithelium and the spermatogenic series (white arrow). Numerous seminiferous tubules in the Obese control group were structurally damaged with a detached seminal epithelium from the basal lamina (red arrow). Cell debris was visible in the lumen of several seminiferous tubules (green arrow). Although signs of testicular degeneration were found in QUE-supplemented obese rats, the damage was less severe and the testicular structure seemed to be improved in comparison to the Obese control. Light microscopy; primary magnification 10× (**a**,**c**,**e**,**g**) and 20× (**b**,**d**,**f**,**h**). Original photos are available as Supplementary Material. Created with (supplementary: Confirmation of Publication and Licensing Rights) BioRender.com (accessed on 9 December 2022).

The testicular tissue of the Lean control group presented with several seminiferous tubules containing irregular basal membranes with signs of disruption in the seminal epithelium and the spermatogenic series (Figure 2a,b). Numerous tubules in the Obese control group were structurally damaged with a detached seminal epithelium from the basal lamina. A severe testicular atrophy accompanied by the disruption of the spermatogenic cycle, denuded spermatogenic cells, and signs of necrosis were observed in numerous cases. Cell debris was present in the lumen of several seminiferous tubules (Figure 2c,d). In the meantime, Lean ZDF rats exposed to QUE were characterized by a predominant presence of normal testicular architecture and regular seminiferous tubular morphology with normal spermatogenesis (Figure 2e,f). The spermatogenic line was undisrupted and contained all the layers of spermatogenic cells. The lumen was filled with spermatozoa. Signs of testicular degeneration were present only sparsely. Although testicular atrophy was found in QUE-administered ZDF obese rats, the number of spermatogenic cells was found to be increased and there was an improvement in the seminiferous tubule structure in comparison with the Obese control (Figure 2g,h). The tissue showed recovery of the histopathological alterations, with the seminiferous tubules containing a higher proportion of the germ cells and a lower abundance of testicular lesions.

**Figure 3:**
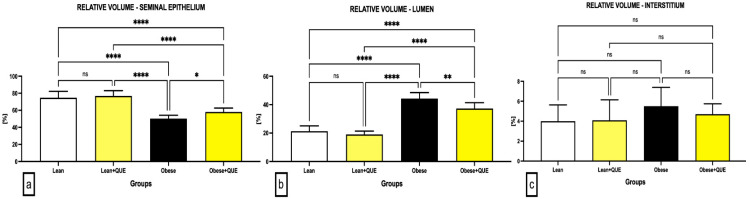
Relative volume of (**a**) the seminal epithelium, (**b**) lumen, and (**c**) interstitium assessed in the testicular tissue of the pre-established control and experimental groups of ZDF rats. Data are presented as mean ± standard deviation. Lean–ZDF lean controls (*n* = 8), Lean + QUE–ZDF lean rats supplemented with QUE (*n* = 9), Obese–ZDF obese controls (*n* = 13), and Obese + QUE–ZDF obese rats supplemented with QUE (*n* = 14). * *p* < 0.05, ** *p* < 0.01, **** *p* < 0.0001, ns—non-significant.

Microscopic observations were furthermore supported by the semi-quantitative analysis of the photomicrographs. A significantly lower (*p* < 0.0001) relative volume of the seminiferous epithelium was found in Obese controls when compared to the Lean control (Figure 3a). QUE administration in ZDF Lean rats led to an insignificant increase in the proportion of the seminal epithelium against the Lean control, whereas a significant increase in the epithelial relative volume was observed in Obese rats subjected to QUE when compared to their controls (*p* < 0.05).

Inversely, the highest relative volume of lumen was recorded in the Obese control, which was significantly higher in comparison to the Lean control (*p* < 0.0001; Figure 3b). A slight non-significant decrease in the luminar relative volume was observed in Lean rats administered with QUE in comparison with the Lean control, while a significant decline in the relative volume of lumen was recorded in Obese rats provided with QUE in comparison with the Obese control (*p* < 0.01). No significant changes were observed in the case of the interstitial relative volume among the pre-established groups, although a decreasing trend was noted when comparing Obese ZDF rats with and without QUE supplementation against each other (Figure 3c).

**Figure 4:**
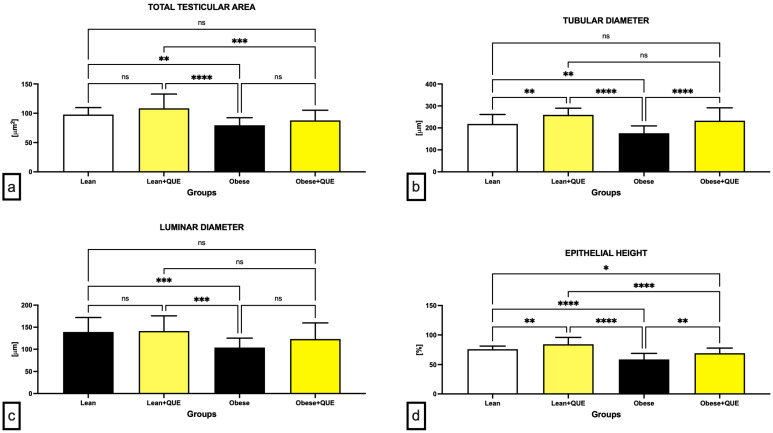
Morphometric characteristics of the testicular tissue including (**a**) the total testicular area, (**b**) tubular diameter, (**c**) luminar diameter, and (**d**) epithelial height of the pre-established control and experimental groups of ZDF rats. Data are presented as mean ± standard deviation. Lean–ZDF lean controls (*n* = 8), Lean + QUE–ZDF lean rats supplemented with QUE (*n* = 9), Obese–ZDF obese controls (*n* = 13), and Obese + QUE–ZDF obese rats supplemented with QUE (*n* = 14). * *p* < 0.05, ** *p* < 0.01, *** *p* < 0.001, **** *p* < 0.0001, ns—non-significant.

Morphometric measurements revealed that in comparison to the Lean control, a significantly decreased total testicular area (*p* < 0.01; Figure 4a), tubular diameter (*p* < 0.01; Figure 4b), luminar diameter (*p* < 0.001; Figure 4c), and epithelial height (*p* < 0.0001; Figure 4d) were present in the Obese control group. QUE supplementation led to an insignificant increase in the total testicular area (Figure 4a) and luminar diameter (Figure 4c) in both Lean as well as Obese rats when compared to their respective controls. On the other hand, a significant increase in the tubular diameter was observed in the Lean + QUE group in comparison to the Lean control (*p* < 0.01) as well as in the Obese + QUE group when compared to the Obese control (*p* < 0.0001; Figure 4b). Similarly, a significant increase (*p* < 0.01) in the epithelial height was observed in both groups exposed to QUE in comparison with their respective controls (Figure 4d).

### 2.2. Oxidative Profile

**Figure 5:**
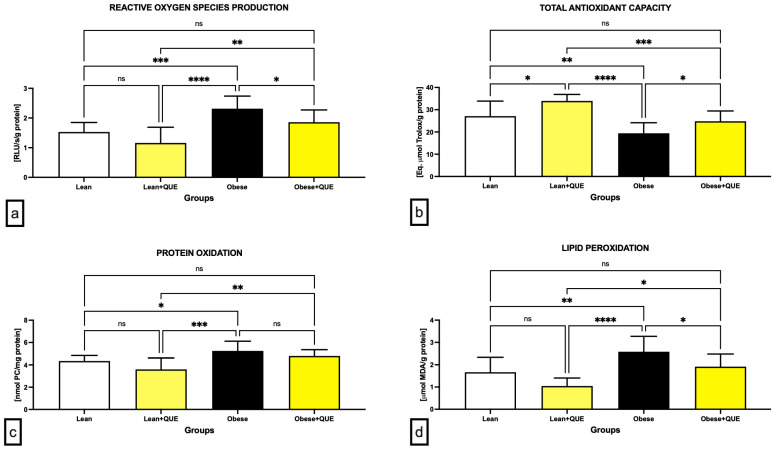
Oxidative profile of the testicular tissue comprising (**a**) reactive oxygen species (ROS) production, (**b**) total antioxidant capacity (TAC), (**c**) oxidative damage to the proteins, and (**d**) oxidative damage to the lipids in the pre-established control and experimental groups of ZDF rats. Data are presented as mean ± standard deviation. Lean–ZDF lean controls (*n* = 8), Lean + QUE–ZDF lean rats supplemented with QUE (*n* = 9), Obese–ZDF obese controls (*n* = 13), Obese + QUE–ZDF obese rats supplemented with QUE (*n* = 14). * *p* < 0.05, ** *p* < 0.01, *** *p* < 0.001, **** *p* < 0.0001, ns—non-significant.

The chemiluminescent analysis revealed a significantly increased ROS production in the Obese control when compared to the Lean control (*p* < 0.001; Figure 5a). While QUE supplementation in the Lean ZDF rats led to an insignificant decrease in ROS in comparison to the Lean control, a significantly decreased ROS production was detected in the testicular tissue of Obese rats that had been administered QUE in comparison to their control counterparts (*p* < 0.05).

Changes in the oxidative milieu in the control and experimental groups were corroborated by a significant decline in total antioxidant capacity (TAC) in the Obese control in comparison with the Lean control (*p* < 0.01; Figure 5b). In the meantime, experimental groups exposed to QUE presented with a significant increase in TAC in comparison to their respective controls (*p* < 0.05).

The highest amount of protein carbonyls indicative of oxidative damage to the proteins was recorded in Obese controls, which was significantly higher when compared to the Lean control (*p* < 0.05; Figure 2c). Although QUE supplementation led to a decrease in oxidized proteins in both experimental groups, this decline was not significant in either case.

Similarly to protein oxidation, significantly higher levels of malondialdehyde (MDA) as the primary marker of lipid peroxidation (LPO) were detected in the Obese control (*p* < 0.01; Figure 5d). While QUE supplementation to Lean animals led to a non-significant decrease in MDA, significantly reduced MDA levels were observed in Obese animals in comparison to their controls (*p* < 0.05).

**Figure 6:**
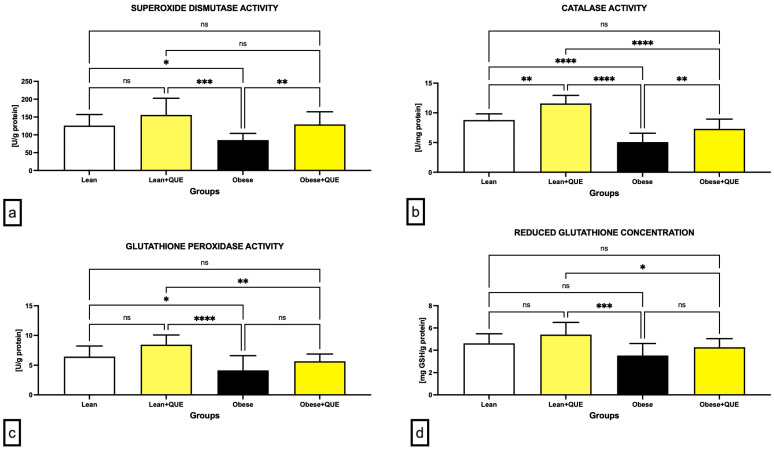
Antioxidant characteristics of the testicular tissue including (**a**) superoxide dismutase activity, (**b**) catalase activity, (**c**) glutathione peroxidase activity, and (**d**) concentration of reduced glutathione in the pre-established control and experimental groups of ZDF rats. Data are presented as mean ± standard deviation. Lean–ZDF lean controls (*n* = 8), Lean + QUE–ZDF lean rats supplemented with QUE (*n* = 9), Obese–ZDF obese controls (*n* = 13), and Obese + QUE–ZDF obese rats supplemented with QUE (*n* = 14). * *p* < 0.05, ** *p* < 0.01, *** *p* < 0.001, **** *p* < 0.0001, ns—non-significant.

A deeper look at the primary components of the antioxidant system of male gonads revealed that superoxide dismutase (SOD) activity was significantly decreased in the Obese control when compared to the Lean group (*p* < 0.05; Figure 6a). As opposed to non-significant changes observed among the Lean control and Lean ZDF rats supplemented with QUE, a significant increase in SOD activity was recorded in Obese rats receiving QUE in comparison to Obese controls (*p* < 0.01).

More pronounced changes were observed in the case of catalase (CAT). Similarly to SOD, activity of the enzyme was the lowest in Obese control rats, which was significantly different in comparison to the Lean control (*p* < 0.0001; Figure 6b). In the meantime, QUE administration to both experimental groups led to a significant increase in CAT activity in comparison with their respective controls (*p* < 0.01). 

Correspondingly, a significant decline in the glutathione peroxidase (GPx) activity was observed in the Obese control when compared to the Lean control group (*p* < 0.05; Figure 6c). Nevertheless, QUE administration led to only an insignificant increase in the enzymatic activity in both Lean as well as Obese ZDF rats when compared with their respective controls.

The levels of glutathione (GSH) considered as a prime non-enzymatic antioxidant present in the testicular tissue did not differ among the controls (Figure 6d). While QUE administration led to an increment in GSH levels in both experimental groups, this trend was not significant in comparison to their respective controls.

### 2.3. Cytokines

**Figure 7:**
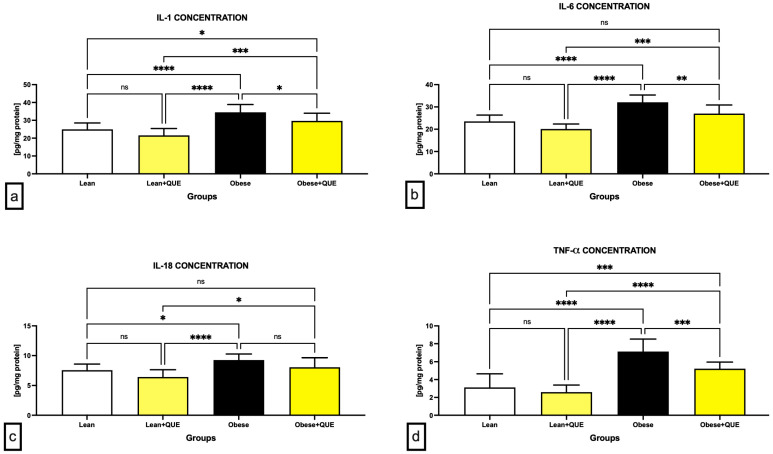
Levels of (**a**) interleukin-1 (IL-1), (**b**) interleukin-6 (IL-6), (**c**) interleukin-18 (IL-18), and (**d**) tumor necrosis factor alpha (TNF-α) in the testicular tissue collected from the pre-established control and experimental groups of ZDF rats. Data are presented as mean ± standard deviation. Lean–ZDF lean controls (*n* = 8), Lean + QUE–ZDF lean rats supplemented with QUE (*n* = 9), Obese–ZDF obese controls (*n* = 13), and Obese + QUE–ZDF obese rats supplemented with QUE (*n* = 14). * *p* < 0.05, ** *p* < 0.01, *** *p* < 0.001, **** *p* < 0.0001, ns—non-significant.

As revealed by the enzyme-linked immunosorbent assay (ELISA), the Obese control presented with significantly higher levels of all cytokines assessed, including interleukin (IL)-1 (Figure 7a), IL-6 (Figure 7b), IL-18 (Figure 7c), and tumor necrosis factor alpha (TNF-α) (Figure 7d) in comparison to the Lean control. QUE supplementation to Lean animals resulted in a non-significant decrease in all cytokines in comparison to their Lean controls. In the meantime, testicular tissues collected from the Obese animals supplemented with QUE presented with significantly decreased levels of IL-1 (*p* < 0.05) as well as IL-6 (*p* < 0.01) in comparison to the Obese control. While a non-significant decrease in IL-18 was recorded in obese animals following QUE administration against the Obese control, a significant decline in TNF-α was recorded in QUE-supplemented Obese animals in comparison with their respective Obese control (*p* < 0.001).

### 2.4. Western Blot

**Figure 8:**
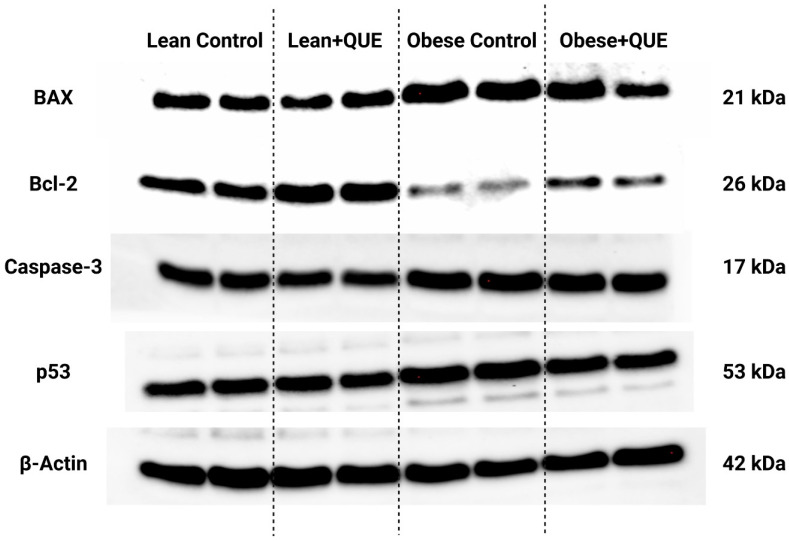
Expression patterns of the BAX, Bcl-2, caspase-3, and p53 protein in the pre-established control and experimental groups of ZDF rats. Data are presented as mean ± standard deviation. Lean–ZDF lean controls (*n* = 8), Lean + QUE–ZDF lean rats supplemented with QUE (*n* = 9), Obese–ZDF obese controls (*n* = 13), and Obese + QUE–ZDF obese rats supplemented with QUE (*n* = 14). The proteins were separated on 4–15% Mini-PROTEAN TGX Stain-Free Protein Gels (BioRad, Hercules, CA, USA). The loading uniformity was confirmed prior to the blotting procedure using the ChemiDoc Imaging System (BioRad, BioRad, Hercules, CA, USA). Respective bands were visualized using appropriate antibodies and ECL-based chemiluminescence. Precision Plus Protein marker (BioRad, Hercules, CA, USA) was used on each gel to indicate the molecular weight of the separated proteins. Original photos are available as Supplementary Material. Created with (supplementary: Confirmation of Publication and Licensing Rights) BioRender.com (accessed on 12 November 2022).

**Figure 9:**
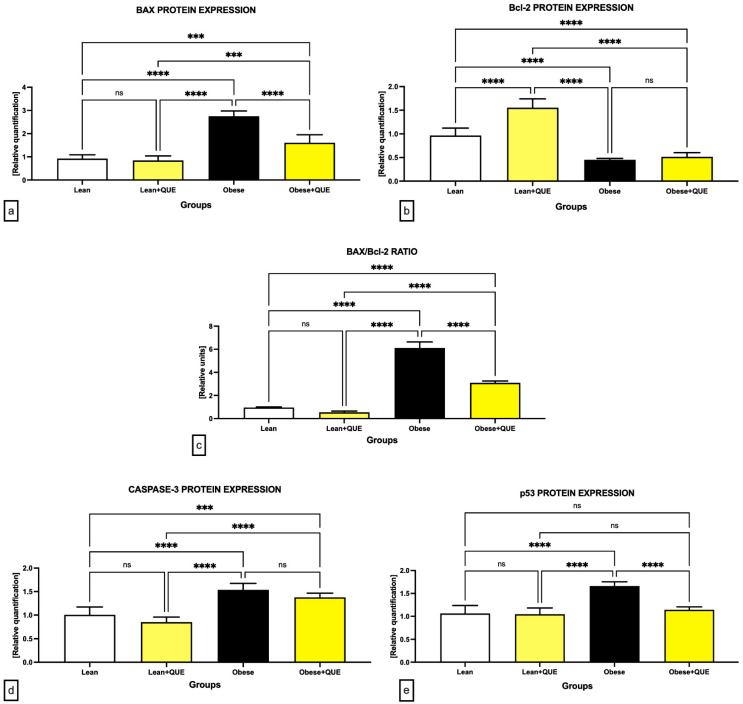
Graphical representation of the relative quantification of the (**a**) BAX, (**b**) Bcl-2, (**d**) caspase-3, and (**e**) p53 protein expression, as well as the BAX/Bcl-2 ratio (**c**) in the pre-established control and experimental groups of ZDF rats. Data are presented as mean ± standard deviation. Lean–ZDF lean controls (*n* = 8), Lean + QUE–ZDF lean rats supplemented with QUE (*n* = 9), Obese–ZDF obese controls (*n* = 13), and Obese + QUE–ZDF obese rats supplemented with QUE (*n* = 14). *** *p* < 0.001, **** *p* < 0.0001, ns—non-significant.

Analysis of the expression patterns of selected proteins involved in apoptosis revealed a significant overexpression of BAX, caspase-3, and p53 in the samples collected from the Obese control in comparison with the Lean control (*p* < 0.0001; Figure 8 and Figure 9). This phenomenon was accompanied by a significantly lower expression of Bcl-2 in Obese animals when compared to their Lean counterparts. Lean ZDF rats supplemented with QUE exhibited a non-significant decrease in all pro-apoptotic proteins accompanied by a significant increase in Bcl-2 (*p* < 0.0001). In the meantime, QUE supplementation to Obese ZDF rats led to a significant decline in the expression of BAX (*p* < 0.0001) and p53 (*p* < 0.0001) with a concomitant non-significant caspase-3 underexpression and a non-significant Bcl-2 overexpression in comparison with the Obese control. Accordingly, the BAX/Bcl-2 ratio was significantly increased in the Obese control when compared to the Lean control. While QUE administration led to a non-significant decrease in the ratio in the Lean experimental group, a significant (*p* < 0.0001) reduction in the BAX/Bcl-2 ratio was observed among the Obese control and Obese ZDF rats supplemented with QUE (Figure 9c).

## 3. Discussion

The present study reveals that Type 2 DM accompanied by obesity causes severe alterations to the testicular architecture that are accompanied by a higher incidence of germ cell apoptosis and disturbance of the inherent oxidative and immunological milieu of male gonads. Inversely, QUE administration provided protection against the impairment of seminiferous tubules and the loss of spermatogenic cell series caused by chronic hyperglycemia and obesity, most likely due to its significant antioxidant, anti-inflammatory, and anti-apoptotic properties. 

A reduced testicular weight in the Obese control observed in this study corroborates previous reports on diabetic rats [22,23,24] and may be explained by the loss of germ cells, inhibition of spermatogenesis, or a decline in the activity of steroidogenic enzymes necessary for a proper sperm production [23,25,26]. In the meantime, the testicular weights were increased following QUE supplementation in both experimental groups. A similar phenomenon was reported by Khaki et al. [27] and Wang et al. [28] hypothesizing that QUE was able to improve the hypothalamic–pituitary–testicular function and ameliorate structural changes to the seminal epithelium caused by chronic hyperglycemia.

Alterations to testicular architecture are a common side effect of chronically elevated blood sugar or excessive accumulation of body fat. In accordance with our morphometric analysis in the untreated animals, previous reports observed that the induction of diabetes through medication or increased feed intake led to a reduced germ cell count and abnormalities to the spermatogenic series [22,23,29,30]. In particular, ZDF obese controls presented with a reduced cell layer with contracted and/or disarranged spermatogenic cells in the seminiferous tubules. The cells were relatively loose, and the resulting sperm density in the lumen was notably lower, which may ultimately compromise reproductive health. Correspondingly to our morphometric outcomes, a recent meta-analysis published by Zhong et al. [8] referred to experimental studies observing a deterioration of the seminiferous epithelium, occurrence of cellular debris, and germ cell detachment from the testicular basal lamina in obese individuals. At the same time, DM has been often accompanied by testicular atrophy and reduction of seminiferous tubules, all of which imply potential spermatogenic deficits [22,30,31]. On the other hand, neither Vendramini et al. [23] nor Mansour et al. [32] observed any significant differences in the morphometric features amongst ZDF Lean and Obese rats. This controversy may be clarified by the fact that both above-mentioned reports employed pubertal or young adult rats, whereas the animals used in our study were of approximately 1 year of age.

Histological alterations to the testicular tissue in this paper were ameliorated by QUE administration. These findings agree with Kanter et al. [17], Ojo and Olorunsogo [18], and Yelumalai et al. [33] who reported that QUE supplementation led to a recovery of the histopathological alterations of the testicular tissue, alongside an improved structure of seminiferous tubules in STZ-induced diabetic rats. QUE was also revealed to support spermatogenesis through stimulation of the testes in otherwise healthy rats via the hypothalamic–pituitary–testicular axis and enlargement of the tubular area, although the extent of such beneficial effects may be directly affected by the dose and time of administration of QUE [34,35]. By and large, all experimental studies published thus far agree that an improvement in testicular architecture in health and disease may be attributed to the strong antioxidant and anti-inflammatory properties of QUE. Collectively, it may be hypothesized that QUE promotes a proper testicular configuration while preventing oxidative insults, chronic inflammation, or excessive cell death of the spermatogenic cell series caused by chronic hyperglycemia and obesity.

Oxidative stress, characterized as a phenomenon when pro-oxidants “overpower” antioxidants, has been acknowledged as a principal hallmark of testicular dysfunction in DM patients [36]. Chronic hyperglycemia may be accompanied by excessive ROS production emerging from increased mitochondrial glucose oxidation, while at the same time, oxidative phosphorylation is slowed down [37]. Excess ROS recorded in this study may subsequently leak out of the mitochondria into the cytoplasm of germ cells, causing oxidative insults to biomolecules critical for spermatogenesis [36,37]. It is well known that male germ cells are more vulnerable to oxidative stress since their membranes contain excessive amounts of easily oxidable polyunsaturated fatty acids [38], providing explanation to the high MDA levels found in the controls. What is more, excessive ROS levels may trigger cell death through the apoptotic machinery [22,39,40], which correlates with our Western blot analyses. Oxidative stress may be aggravated in the presence of excessive body fat, as observed in our study. Obesity is associated with chronic inflammation, high cellular metabolism, and a consequent release of reactive intermediates into the testicular tissue. ROS overgeneration may also be caused by the action of pro-inflammatory cytokines and phagocytes intrinsically predisposed to release ROS during oxidative bursts [41]. Changes in the levels of antioxidant molecules, particularly in the Obese control, indicate a shift in the oxidative homeostasis, which may be caused by increased intracellular ROS levels and a higher degree of oxidative damage to the germ cells [37,42]. Moreover, this disproportion may be accompanied by depletion of the inherent antioxidant capacity, which agrees with previous studies [17,18,19,22,27]. The inability of SOD to dismutate superoxide to hydrogen peroxide (H_2_O_2_), followed by a decreased capacity of CAT or GPx to break H_2_O_2_ down to water and oxygen, may lead to the accumulation of H_2_O_2_ and other toxic reactive intermediates, with a subsequent loss of proteins and lipids [43]. As such, we may speculate that enzymatic antioxidants are particularly prone to deterioration, leading to the buildup of ROS within testicular structures in subjects suffering from Type 2 DM. 

In the meantime, QUE administration led to a notable stabilization of the testicular oxidative profile, more so in obese animals. Within flavonoids, QUE tends to act as an exceptional scavenger of ROS and nitric oxide [10], which was corroborated by our results. Moreover, QUE has been reported to contribute to the endogenous antioxidant capacity of male reproductive tissues, particularly by stabilizing the levels of antioxidant enzymes [17,19,27]. Interestingly, out of the enzymes responsible for H_2_O_2_ detoxication, CAT seemed to respond more to QUE treatment, while no significant differences were observed in the case of GPx. This is in disagreement with Kanter et al. [17] who noted a significant GPx recovery in male rats treated intraperitoneally with 15 mg QUE/kg. Furthermore, a significant restoration of thiols was observed by Hajiyeva et al. [19] in diabetic rats exposed to 20 mg QUE/kg/day intraperitoneally, while we recorded only an insignificant rise in GSH levels in the experimental groups. While CAT activity was not assessed in either of the above-referenced studies, the discrepancies among our data and previous reports may have been caused by a different experimental DM model, dose, and administration mode of QUE to the animals. Regardless of the experimental design, all previous papers agree with our data indicating a strong potential of QUE to prevent excessive damage to lipids [17,18,19,27]. This phenomenon may be caused by the inherent ability of QUE to interact with and penetrate lipid bilayers of the germ cell membranes [10]. We may speculate that once QUE has entered the membranes, its 3′-O (C3-hydroxyl group) and 9′-O (C9-hydroxyl group) positions are available for ROS. As such, by its direct ROS-quenching ability, QUE may decrease the concentration of ROS disposed to cause damage to testicular tissue. Furthermore, the ability of QUE to stabilize the levels of antioxidant enzymes enables ROS-detoxication reactions to occur, preventing eventual ROS accumulation and formation of lipid peroxides. 

Metabolic syndrome arising from DM and obesity may be defined as a cluster of conditions that may promote the secretion of cytokines predictive of insulin resistance [44,45]. As unraveled by earlier reports, chronic hyperglycemia is associated with the activation of two major pro-inflammatory molecular pathways, namely the transcription factor NF-κB pathway (NF-κB) and the stress-activated Jun N-terminal kinases (JNK) pathway [46]. Both pathways will promote the secretion of inflammatory cytokines, which may be additionally stimulated by the activity of adipokines produced by the body fat [47]. Within this heterogenous group of cell-signaling molecules, TNF-α, interleukins, adiponectin, leptin, resistin, or chemokines may become involved in inflammation and further disturbance to blood sugar regulation [41,46,47].

In this study, we focused on four major cytokines that have been suggested to play an important role in male reproductive dysfunction, specifically IL-1, IL-6, IL-18, and TNF-α. As anticipated, all selected pro-inflammatory molecules were found to be increased in the Obese control. Accordingly, high levels of IL-1, IL-6, and TNF-α have been observed during a local or systemic inflammation, directly affecting testicular steroidogenesis and spermatogenesis [33,48]. Within the testicular tissue, these molecules may directly interfere with the expression patterns of the cytoskeletal and junctional proteins, and thus cause openings in the cell junctions between Sertoli and epithelial cells, leading to disturbances in the niche of the seminiferous epithelium critical for proper spermatogenesis [49,50]. While little is known about the exact function of IL-18, it has been reported that the molecule is highly expressed in autoimmune diseases [51] and may accelerate the production of anti-sperm antibodies that are a typical consequence of male subfertility caused by a systemic inflammation [52]. Furthermore, it has been suggested that IL-18 could be involved in the control of testicular cell proliferation and apoptosis, which supports the highest expression of pro-apoptotic proteins in the samples with the highest levels of IL-18 found in this study. Finally, it has been also acknowledged that high IL-18 activity could directly inhibit spermatogenesis through an enhanced oxygen metabolism, and thus lead to a higher probability of ROS overproduction [53].

Both in vitro and in vivo studies suggest that QUE ameliorates inflammation either by a direct inhibition of the production of inflammatory mediators, including ROS and reactive nitrogen species (RNS), or through preventing the release of pro-inflammatory cytokines, such as TNF-α and IL-1. Several research papers have suggested that the anti-inflammatory effect of QUE may be associated with a down-regulation of NF-κB activity, although without significantly affecting the JNK signaling pathway [54,55]. While the exact mechanism of action of QUE on NF-κB suppression is yet to be elucidated, the currently available body of evidence suggests that the biomolecule may inhibit NF-κB through its direct antioxidant properties, since NF-κB is a redox-sensitive transcription factor triggered by oxidative stress to begin with [56]. A similar pattern of QUE behavior was also observed by Yelumalai et al. [33], who reported on significantly decreased NF-κB expression patterns in spermatozoa collected from STZ-nicotinamide-induced diabetic rats supplemented orally with 10–50 mg QUE/kg/day. The authors speculated that QUE treatment affects the translocation of NF-κB from the cytoplasm into the nucleus, leading to changes in the synthesis of pro-inflammatory genes. Moreover, it has been suggested that QUE inhibits nitric oxide synthase expression and subsequent nitric oxide release in activated macrophages and neutrophils [57,58]. 

In line with our results, previous papers have also observed that QUE administration could remarkably decrease TNF-α during inflammatory processes. This reduction may be caused by the ability of QUE to interact with myeloperoxidase (MPO) found in neutrophils, which is directly involved in the generation of excessive ROS causing tissue damage [59]. QUE has been demonstrated to down-regulate the activity of MPO in hypercholesterolemic rats, which was accompanied by a decreased neutrophil infiltration and a reduced ROS generation [60]. Furthermore, in in vitro experiments by Pečivová et al. [61], exposure of human neutrophils to QUE led to a reduced MPO activity and ROS release. QUE was also reported to inhibit the productions of pro-inflammatory cytokines, including IL-1 and IL-6, from activated macrophages [62]. Similarly to this study, ameliorative effects of QUE have also been observed in subjects suffering from chronic inflammatory diseases through the reduction of selected inflammatory markers, such as TNF-α or IL-8 [63].

Regardless of growing options to manage DM-associated male subfertility, excessive testicular cell death remains a major challenge in this process. Apoptosis is an essential regulator of physiological events; nevertheless, its up-regulation can significantly contribute to the onset and progression of a wide array of diseases [64]. Our Western blot data collected from untreated controls agree with previous immunocytochemistry-based studies on diabetic rats [18,19,22]. It has been suggested that a persistent hyperglycemia causes alterations to the proapoptotic BAX and the antiapoptotic Bcl-2 protein, while simultaneously up-regulating p53, caspase-8, and caspase-9. All these events will ultimately result in the activation of caspase-3 as the prime executor of apoptotic cell death [65,66]. Correspondingly to caspase-3 overexpression recorded in this study, Nna et al. [42] observed caspase-3 mRNA as well as protein up-regulation, which correlated with an abrupt loss of testicular cells in STZ-induced diabetic rats. Similarly, He et al. [67] commented on a significant caspase-3 positive immunocytochemical signal located primarily in Sertoli cells, spermatogonia, and spermatocytes of rats presenting with a severe hyperglycemia caused by STZ and a high fat diet. According to Ojo and Olorunsogo [18], who observed an increased release of cytochrome-c in diabetic rats, testicular apoptosis is primarily regulated by the mitochondrial pathway which corroborates our data on the BAX and Bcl-2 proteins whose interactions on the mitochondrial membrane have a decisive role in the initiation of apoptotic cell death. Finally, Kanter et al. [17] revealed that the signal density of the proliferating cell nuclear antigen (PCNA) indicative of mitotic testicular epithelium proliferation was significantly decreased in STZ-induced diabetic rats.

The ability of QUE to interfere with the apoptotic machinery has been reported in several cell types. In general, QUE facilitates apoptosis of tumor cells, while it may inhibit cell death in some non-tumorigenic cells, such as fibroblasts, mesangial, or epithelial cells [68]. Based on our results, QUE has the potential to modulate the expression patterns of molecular markers involved in apoptosis, leading to a higher preservation of testicular germ cells. Accordingly, Ojo and Olorunsogo [18] showed that QUE alone or in combination with vitamin E improved excessive cytochrome c release, thus reducing the recruitment of the apoptotic protease activating factor 1 (Apaf-1) and apoptosome required for the activation of caspase-9. Since it is acknowledged that activation of caspase-9 triggers caspase-3, Ojo and Olorunsogo [18] speculate that QUE administration would reduce excessive caspase-3 activity and thus down-regulate testicular death in diabetes. This hypothesis was subsequently confirmed by later experimental studies [19,22,28] and is supported by our results as well. 

We must acknowledge that this study presents several limitations. There could have been another diabetic group of animals that would present with symptoms consistent with the onset and/or progress of Type 1 DM. This may have enabled us to follow the similarities and differences of the molecular response to QUE supplementation amongst the two most prevalent DM types. Furthermore, we may have used a broader range of QUE concentrations to understand beneficial as well as toxic effects of QUE on the testicular tissue in sickness and health, since evidence on the impact of QUE on male reproduction is still controversial. Additionally, the assessment of a larger spectrum of inflammation and apoptotic markers might have provided more clarity on the specific mechanism of action of QUE on male gonads. Finally, an important limitation to the exploitation of the full potential of QUE lies in its reduced oral bioavailability [69]. While the biomolecule is able to easily enter the cells through the phospholipid bilayer [70], it has poor aqueous solubility and is instable in physiological media [69]. As such, its absorption in the digestive tract is reduced since the intestinal cells are surrounded by a mucus layer with 90% water content [71]. If the mucus barrier is trespassed, QUE is rapidly metabolized in the liver and blood via glucuronidation, methylation, and sulfation [72], which is why glucuronic acid, sulfate, or methyl conjugates are found in blood as opposed to QUE aglycone [73]. This opens the question of whether metabolites derived from QUE would be more suitable for therapeutic intervention; nevertheless, it has been revealed that the in vivo activity major QUE-derived metabolites such as QUE glucosides are much weaker than QUE itself [74] or QUE aglycone. As such, scientific effort has switched towards novel vehicles to improve oral absorption and bioavailability of QUE, out of which micelles, liposomes, and nanosuspensions show promising preliminary results [69,71]. In this study, we elected a more conservative approach to administer QUE via biscuits, which are conveniently prepared without the necessity for sophisticated equipment. Furthermore, the biscuits are easily consumed by the rats without additional distress that may occur in the case of gastric gavage. We may speculate that QUE bioavailability might have been higher in the biscuits when compared to a water-based QUE solution, since the biomolecule may have interacted with the carbohydrates and fats present in the vehicle which could have facilitated its entry to the intestinal cells. Nevertheless, we did not evaluate the presence and/or concentration of QUE or its metabolites in blood, which is a key endeavor to be researched in the future.

## 4. Materials and Methods

### 4.1. Animals and Treatment

This study employed one-year-old male ZDF (fa/+) Lean rats (*n* = 17), and their ZDF (fa/fa) Obese counterparts (*n* = 27), that were obtained from the breeding facility at the Department of Toxicology and Laboratory of Animal Breeding, Centre of Experimental Medicine (Slovak Academy of Sciences, Dobrá Voda, Slovakia). The animals were housed in plastic cages at a stable temperature of 22 ± 2 °C, with a 12 h light/12 h dark photoperiod regime and air humidity of 45–65%. The rats were provided with KZ-*p*/M chow (complete feed mixture for rats and mice, reg. no 6147; Dobrá Voda, Slovakia) and drinking water was available ad libitum. Feed and water consumption as well as body weights of the animals were checked every other day [69].

The animals were distributed into 4 experimental groups: vehicle-treated Lean ZDF control (“Lean”; *n* = 8), QUE-treated Lean ZDF rats (“Lean + QUE”; *n* = 9), vehicle-treated Obese ZDF control (“Obese”; *n* = 13), and QUE-treated Obese ZDF rats (“Obese + QUE”; *n* = 14). QUE (#Q4951, ≥95% purity; Sigma-Aldrich, St. Louis, MO, USA) was dissolved in ethanol (Sigma-Aldrich, St. Louis, MO, USA) and provided in a biscuit (vehicle) at a concentration of 20 mg/kg/day for 6 weeks [75,76]. For the Lean and Obese controls, the biscuit contained a small amount of ethanol. The length of treatment, route of administration, and QUE dose were selected upon previous experiments published by this group [77]. The biscuits were prepared from wheat flour (T-450; 53.80%), eggs (42.70%), and granulated sugar (3.50%) purchased in a local supermarket. All ingredients were mixed together, and the resulting dough was baked at 170 °C for 15 min. After cooling for 30 min, the dough was cut into small biscuits of equal weight which were air-dried. Subsequently, the biscuits were packed in polyethylene zipper food plastic bags and stored at +21 °C and 50% relative humidity. The biscuits were provided to the rats every day in the morning.

To confirm diabetes in the animals, fasting blood glucose levels were measured in a blood drop collected from the tail vein shortly before QUE treatment had started and the end of the administration period of QUE. Systolic and diastolic blood pressure and heart rate were also monitored at the beginning and end of QUE treatment by the non-invasive method of tail cuff plethysmography [76]. Subsequently, the animals were sacrificed using thiopental (50 mg/kg, intraperitoneally) and heparin (500 IU, subcutaneously). To verify changes in the lipid profile as a result of diabetes, total cholesterol, low density lipoprotein-cholesterol, high density lipoprotein-cholesterol, and total triglycerides were measured in blood plasma. The measurements revealed significant changes in the body weight among the Lean and Obese control (*p* < 0.0001) that were accompanied by significantly increased levels of glucose, triglycerides, total cholesterol, high density lipoprotein-cholesterol (*p* < 0.0001), and systolic blood pressure (*p* < 0.01) in the Obese control when compared to the Lean control, that are changes consistent with the onset of Type 2 DM. Numerical data and specific comments to the above-mentioned measurements are provided by Ferenczyova et al. [76].

**Figure 10:**
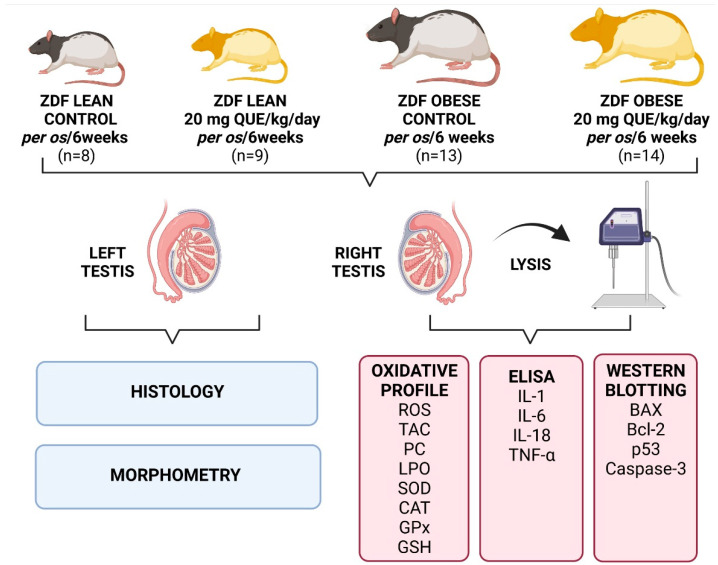
Overview of the experimental approach. ROS—reactive oxygen species, TAC—total antioxidant capacity, LPO—lipid peroxidation, SOD—superoxide dismutase, CAT—catalase, GPx—glutathione peroxidase, GSH—glutathione, IL-1—interleukin-1, IL-6—interleukin-6, IL-18—interleukin-18, TNF-α—Tumor necrosis factor alpha. Created with (supplementary: Confirmation of Publication and Licensing Rights) BioRender.com (accessed on 9 December 2022).

Immediately following animal sacrifice, male reproductive organs were excised from the scrotum, testes were separated from the epididymides, weighed, and processed further. A resume of the experimental outline is provided in Figure 10.

### 4.2. Histology and Morphometry

Left testes were fixed using 10% formaldehyde (Centralchem, Bratislava, Slovakia), dehydrated in a grade series of ethanol (Centralchem, Bratislava, Slovakia), saturated with benzene (Centralchem, Bratislava, Slovakia), and embedded with paraffin (Centralchem, Bratislava, Slovakia). Paraffin blocks were then sectioned into 5 μm thick sections which were fixed onto microscopic slides and stained with hematoxylin and eosin (Sigma-Aldrich, St. Louis, MO, USA). Photomicrographs were taken at a magnification of 10× and 20× using the Leica LAS EZ software and optical microscopy (EC3 microscope, Leica Camera AG, Wetzlar, Germany). Morphometric evaluation of the testicular tissue was carried out with the QuickPHOTO MICRO program (Promicra, Prague, Czech Republic) as previously described by Tvrda et al. [22].

### 4.3. Tissue Lysis

Right testes were cut into small fragments of approximately 50 mg which were treated with the RIPA buffer/protease inhibitor cocktail (Sigma-Aldrich, St. Louis, MO, USA) and lysed using the SFX 250 ultrasonic homogenizer (Branson Ultrasonics, Brookfield, CT, USA) on ice at 28 kHz for 30 s. Following centrifugation (11,828× *g*, 4 °C, 15 min), the lysates were separated from the cell debris and subjected to protein quantification [22]. The Total protein commercial kit (DiaSys Diagnostic Systems, Holzheim, Germany) was used to process each tissue lysate, and protein concentration was measured with the Rx Monza semi-automatic analyzer (Randox Laboratories Ltd., Crumlin, UK) [22]. Finally, the lysates were stored at −80 °C until further analysis.

### 4.4. Oxidative Profile Assessment

ROS levels in each tissue lysate were quantified with the luminol-based chemiluminescent assay. The specimens were mixed with 2.5 μL 5 mmol/L luminol (5-amino-2,3-dihydro-1,4-phthalazinedione; Sigma-Aldrich, St. Louis, MO, USA) and subjected to chemiluminescent assessment using the Glomax Multi+ combined spectro-fluoro-luminometer (Promega Corporation, Madison, WI, USA). Each measurement involved a positive control (100 μL phosphate buffer, 2.5 μL luminol, and 12.5 μL 30% H_2_O_2_; Sigma-Aldrich, St. Louis, MO, USA) and a negative control (100 μL PBS). The results are expressed as relative light units (RLU)/s/g protein [22].

The sum of all antioxidants present in the samples defined as the total antioxidant capacity (TAC) was quantified using an improved chemiluminescent assay introduced by Muller et al. [78]. Briefly, the samples were treated with a signal reagent containing 282.2 mmol/L luminol, 41.8 mmol/L 4-iodophenol (Sigma-Aldrich, St. Louis, MO, USA), 12 mol/L H_2_O_2_, and 0.4% (*v*/*v*) horseradish peroxidase (Sigma-Aldrich, St. Louis, MO, USA), and the resulting chemiluminescent signal was monitored during 10 consecutive 1-min-long cycles using the Glomax Multi+ combined spectro-fluoro-luminometer. The results were calculated with the help of a pre-established Trolox (6-hydroxy-2,5,7,8-tetramethylchroman-2-carboxylic acid; 5–100 μmol/L; Sigma-Aldrich, St. Louis, MO, USA) standard curve and are quoted as Eq. μmol Trolox/g protein [22,78].

The amount of protein carbonyls (PC) indicative of oxidative damage to proteins was assessed with the help of a traditional 2,4-dinitrophenylhydrazine (DNPH) method developed by Weber et al. [79]. In brief, each specimen was normalized to 1 mg protein/mL, precipitated with trichloroacetic acid (TCA; 20% *w*/*v*; Sigma-Aldrich, St. Louis, MO, USA), subsequently treated with 1 mL DNPH (10 mM in 2 N HCl; Sigma-Aldrich, St. Louis, MO, USA) and incubated at 37 °C for 1 h. Following incubation, fresh 20% TCA was added to the samples, which were then centrifuged (300× *g*, 15 min). The obtained protein pellets were washed three times with 1 mL of ethanol/ethyl acetate (1/1; *v*/*v*; Sigma-Aldrich, St. Louis, MO, USA) and resuspended in 1 mL 6 mol/L guanidine hydrochloride (Sigma-Aldrich, St. Louis, MO, USA). Absorbance measurement was carried out with the Cary 60 UV-Vis spectrophotometer (Agilent Technologies, Santa Clara, CA, USA) at 360 nm, and protein carbonylation was calculated using the molar absorption coefficient of 22,000 1/M.cm. The results are expressed as nmol PC/mg protein [22,80].

The extent of LPO, expressed through MDA levels, was assessed with the thiobarbituric acid reactive substances (TBARS) assay, modified for a 96-well plate. Each sample was pretreated with 5% sodium dodecyl sulfate (Sigma-Aldrich, St. Louis, MO, USA), subsequently exposed to 0.53% thiobarbituric acid (Sigma-Aldrich, St. Louis, MO, USA) in 20% acetic acid (Centralchem, Bratislava, Slovakia), and boiled at 100 °C for 1 h. To stop the ongoing reaction, the samples were placed on ice for 10 min and centrifuged at 1750× *g* for 10 min. Supernatants were collected, transferred (150 μL) to a 96-well plate, and the resulting absorbance was measured at 540 nm using the Glomax Multi+ combined spectro-fluoro-luminometer [22,80]. The results were calculated with the help of a standard curve and are quoted as μmol MDA/g protein.

Commercial kits RANSOD (Randox Laboratories, Crumlin, UK) and RANSEL (Randox Laboratories, Crumlin, UK) were used for the assessment of SOD and GPx activity, respectively. The samples were processed following the instructions of the manufacturer, and activities of both enzymes were measured with the Rx Monza semi-automatic analyzer. The results are expressed as IU/g protein [22,80]. CAT activity was assessed using the methodology established by Beers and Sizer [81] that is based on tracking H_2_O_2_ decline at 240 nm using the Cary 60 UV-Vis spectrophotometer. The obtained data are quoted as IU/mg protein [22,80].

Levels of reduced GSH were evaluated by the Ellman method [82], which is based on sample precipitation using 10% TCA/10 mmol/L EDTA (pH 8.2; Sigma-Aldrich, St. Louis, MO, USA) and a subsequent treatment with 10 mmol/L DTNB (5,50-dithiobis-(2-nitrobenzoic acid); Ellman’s reagent; Sigma-Aldrich, St. Louis, MO, USA). The resulting colorimetric reaction was monitored at 412 nm with the help of the Genesys 10 spectrophotometer (Thermo Fisher Scientific, Waltham, MA, USA). GSH levels were calculated using a standard curve and are quoted as mg GSH/g protein [22,80].

### 4.5. Cytokine Analysis

IL-1, IL-6, IL-18, and TNF-α levels were assessed using commercial ELISA kits suitable for rat tissue lysates (#RAB0278, #RAB0312, #RAB1147, and #RAB0480, respectively; Sigma-Aldrich, St. Louis, MO, USA). All assays are based on a double-sandwich procedure and were performed as per the instructions of the manufacturer. Concentrations of the selected cytokines were measured with the Glomax plate spectrophotometer (Promega Corporation, Madison, WI, USA) at 450 nm and are expressed as pg/mg protein [22].

### 4.6. Western Blotting

Prior to the assay, all lysates were normalized, i.e., protein concentration was adjusted using ultrapure (UHQ) water to reach the final concentration of 25 μg protein. The samples were treated with 4× Laemli buffer (BioRad, Hercules, CA, USA), β-mercaptoethanol (Sigma-Aldrich, St. Louis, MO, USA), and subsequently boiled at 95 °C for 10 min.

The pre-treated samples were loaded (20 μL) into Mini-PROTEAN TGX Stain-free polyacrylamide gels (BioRad, Hercules, CA, USA), along with 7 μL of Precision Plus Protein marker (BioRad, Hercules, CA, USA). Gel electrophoresis was run at 90 V for 2 h; subsequently, the gel was visualized with the ChemiDoc Imaging System (BioRad, Hercules, CA, USA). For the blotting procedure, the gels were transferred to PVDF membranes (Trans-Blot Turbo Pack; BioRad, Hercules, CA, USA) using the Trans-Blot Turbo Transfer System (BioRad, Hercules, CA, USA), at 7 min, 25 V, and 2.5 A. After completion of the blot, the sandwich was disassembled, and the membranes were incubated for 3 × 10 min in tris buffered saline (TBS), composed of Tris base (Sigma-Aldrich, St. Louis, MO, USA), sodium chloride (Centralchem, Bratislava, Slovakia), and UHQ water. This step was followed by membrane blocking on a stirrer at room temperature for 2 h. Subsequently, the membranes were treated with primary antibodies against the proteins of interest overnight at 4 °C. Antibodies used for the detection of target proteins are specified in Table 1.

The next day, the membranes were washed 5 × 10 min in wash buffer containing 1% milk (BioRad, Hercules, CA, USA) in TBS/0.2% Tween-20, and subsequently incubated with a secondary anti-rabbit antibody (GE Healthcare, Chicago, IL, USA) diluted 1:15,000 in 1% milk in TBS/0.2% Tween-20 for 1 h. Following incubation, the membranes were washed 5 × 10 min in TBS/0.2% Tween-20 at room temperature and using a stirrer.

To visualize the target proteins, membranes were incubated with the ECL substrate (GE Healthcare, Chicago, IL, USA) in the dark for 5 min. After incubation the membranes were placed on the ChemiDoc Imaging System, which automatically calculated the protein visualization time based on the light signal emitted by the membranes.

Relative quantification of the protein expression was assessed using BioRad Image Software 6.1 (BioRad, Hercules, CA, USA). For all the blots, the expression of a housekeeping protein, e.g., β-actin was assessed (Table 1) [83]. The results are interpreted as relative quantification of the proteins against the Lean control.

### 4.7. Statistics

Statistical analysis was carried out with the GraphPad Prism program (version 8.4.4 for Mac; GraphPad Software Incorporated, La Jolla, CA, USA). The results are expressed as mean ± standard deviation. Firstly, the data were processed with the Shapiro–Wilk normality test taking a normal (Gaussian) distribution into consideration. All data sets passed the test with non-significant results at the alpha level of 0.05. Differences between the groups were analyzed using one-way ANOVA followed by the Tukey multiple comparison test, designed to compare the means of three or more independent samples simultaneously. Statistical significance was set at *p* < 0.05 (*), *p* < 0.01 (**), *p* < 0.001 (***) and *p* < 0.0001 (****).

## 5. Conclusions

In conclusion, we may suggest that QUE exhibits protective effects on testicular tissue compromised by Type 2 diabetes mellitus, particularly by its ability to stabilize intracellular antioxidant defense systems and to reduce the extent of oxidative damage to testicular cells and tissues. Furthermore, the molecule was revealed to possess significant anti-inflammatory and anti-apoptotic properties, which may be useful for the development of new treatment options for subfertile patients suffering from diabetes and/or obesity. Nevertheless, further studies on the effects of QUE on the process of steroidogenesis and spermatogenesis in health and disease are needed.

## Figures and Tables

**Table 1:** Antibodies used in the Western blot analysis.

Target Protein	Antibody	Clonality/Isotype	Dilution	Blocking Solution	Source	ID	Manufacturer
BAX	anti-BAX antibody (BCL2-Associated X Protein) N-Term	Polyclonal/IgG	1:1000	5% milk in TBS/0.1% Tween-20	rabbit	#ABIN6990475	Antibodies Online; Dunwoody, GA, USA
Bcl-2	anti-Bcl-2 antibody (B-Cell CLL/lymphoma 2) N-Term	Polyclonal/IgG	1:1000	5% milk in TBS/0.1% Tween-20	rabbit	#ABIN2857047	Antibodies Online; Dunwoody, GA, USA
p53	anti-p53 antibody [PAb421]	Monoclonal/IgG	1:1000	5% milk in TBS/0.1% Tween-20	rabbit	#ab245685	Abcam, Cambridge, UK
Caspase-3	Caspase 3 (Cleaved Asp175)	Polyclonal/IgG	1:1000	5% milk in TBS/0.1% Tween-20	rabbit	#PA5-114687	Invitrogen, Waltham, MA, USA
β-actin	beta Actin Polyclonal Antibody	Polyclonal/IgG	1:1000	5% milk in TBS/0.1% Tween-20	rabbit	#PA1-16889	Invitrogen, Waltham, MA, USA

## Data Availability

The datasets generated during and/or analyzed in this study are available from the corresponding author upon reasonable request.

## Supplementary Materials

The following supporting information can be downloaded at: https://www.mdpi.com/article/10.3390/ijms232416056/s1, Figure S1: Original photomicrograph of Figure 2a; Figure S2: Original photomicrograph of Figure 2b; Figure S3: Original photomicrograph of Figure 2c; Figure S4: Original photomicrograph of Figure 2d; Figure S5: Original photomicrograph of Figure 2e; Figure S6: Original photomicrograph of Figure 2f; Figure S7: Original photomicrograph of Figure 2g; Figure S8: Original photomicrograph of Figure 2h; Figure S9: Original blot of BAX protein; Figure S10: Inverted blot of BAX protein; Figure S11: Original blot of Bcl-2 protein; Figure S12: Inverted blot of Bcl-2 protein; Figure S13: Original blot of p53 protein; Figure S14: Inverted blot of p53 protein; Figure S15: Original blot of caspase-3 protein; Figure S16: Inverted blot of caspase-3 protein; Figure S17: Original blot of beta actin; and Figure S18: Inverted blot of beta actin.
