# Supplementary material for: Quercetin Ameliorates Testicular Damage in Zucker Diabetic Fatty Rats through Its Antioxidant, Anti-Inflammatory and Anti-Apoptotic Properties
Source: Int J Mol Sci. 2022 Dec 16;23(24):16056. doi: 10.3390/ijms232416056 (PMC9781092; doi:10.3390/ijms232416056)
Supplement: Supplementary file 1 [file ijms-23-16056-s001.zip › ijms-2076882-supplementary.pdf]

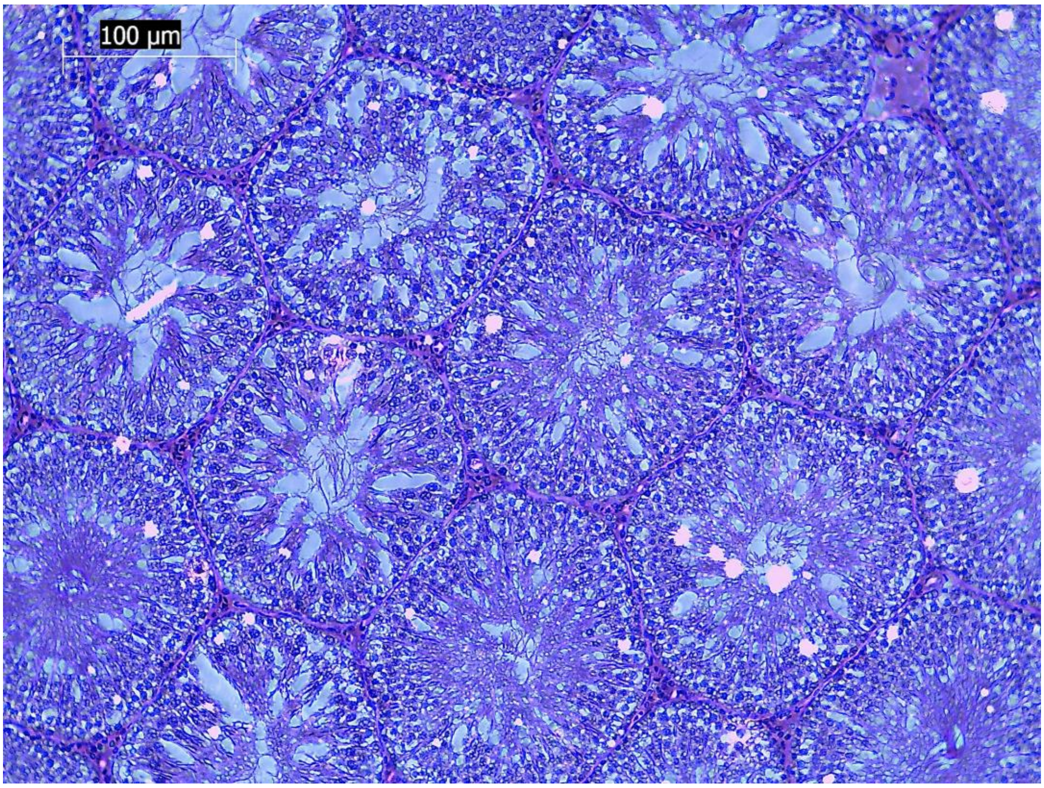

Figure S1. Original photomicrograph of Figure 2a.

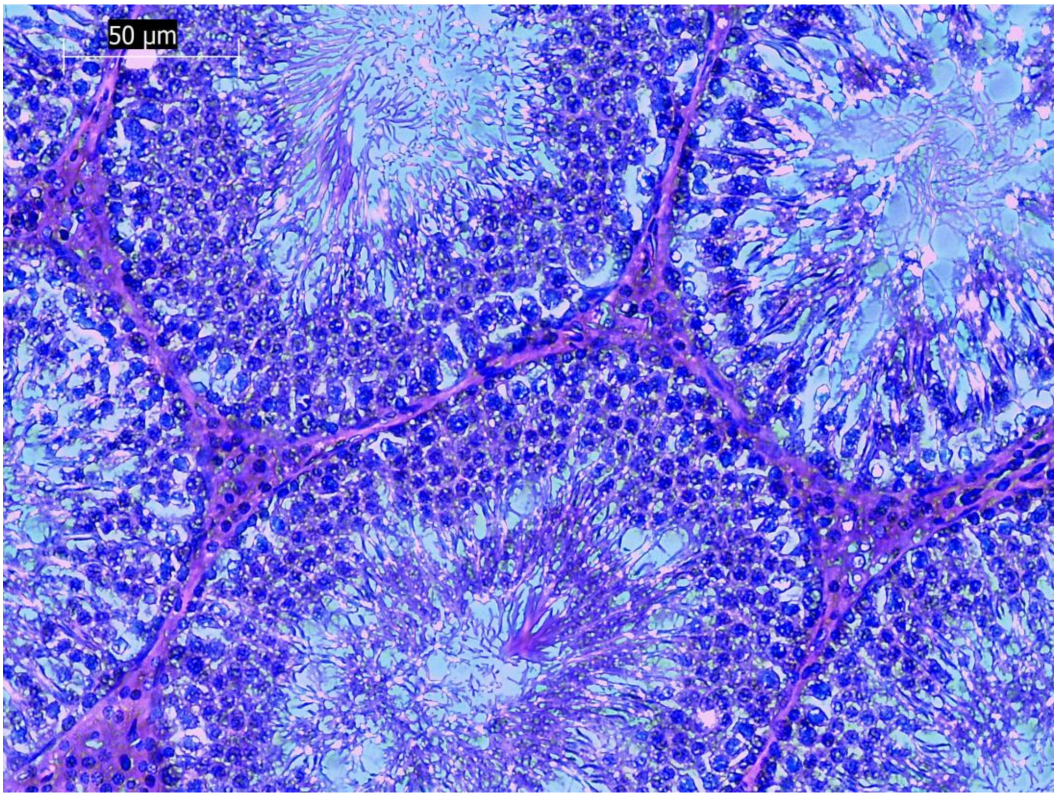

Figure S2. Original photomicrograph of Figure 2b.

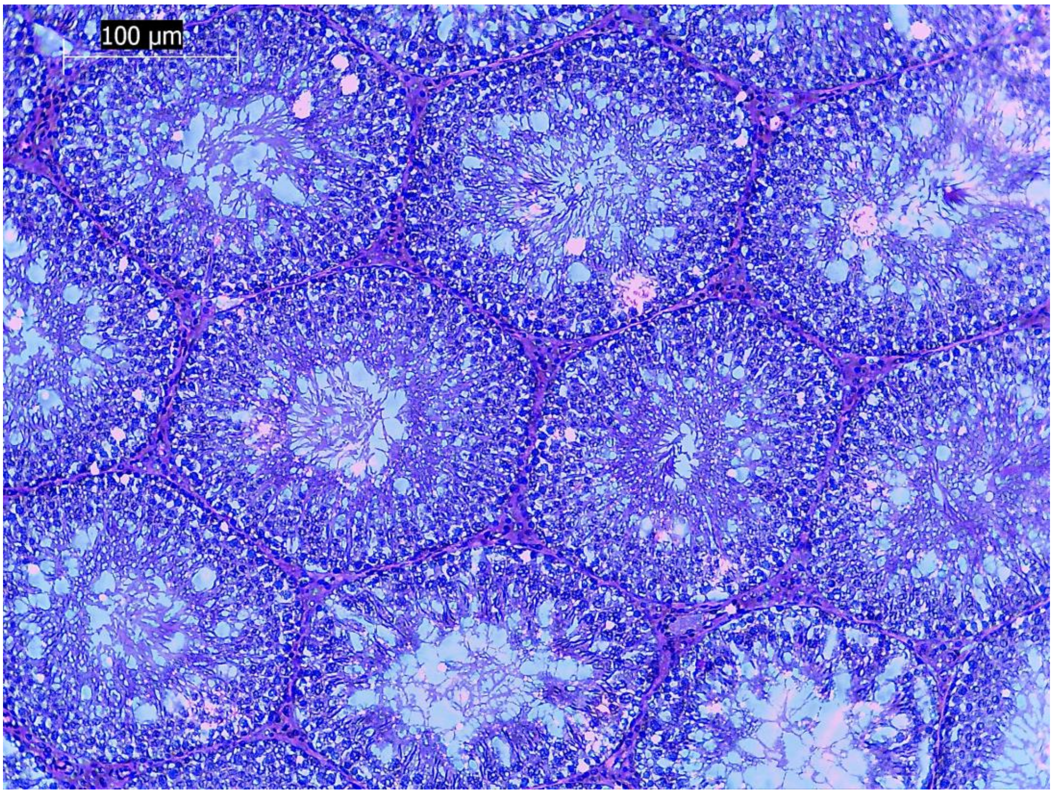

Figure S3. Original photomicrograph of Figure 2c.

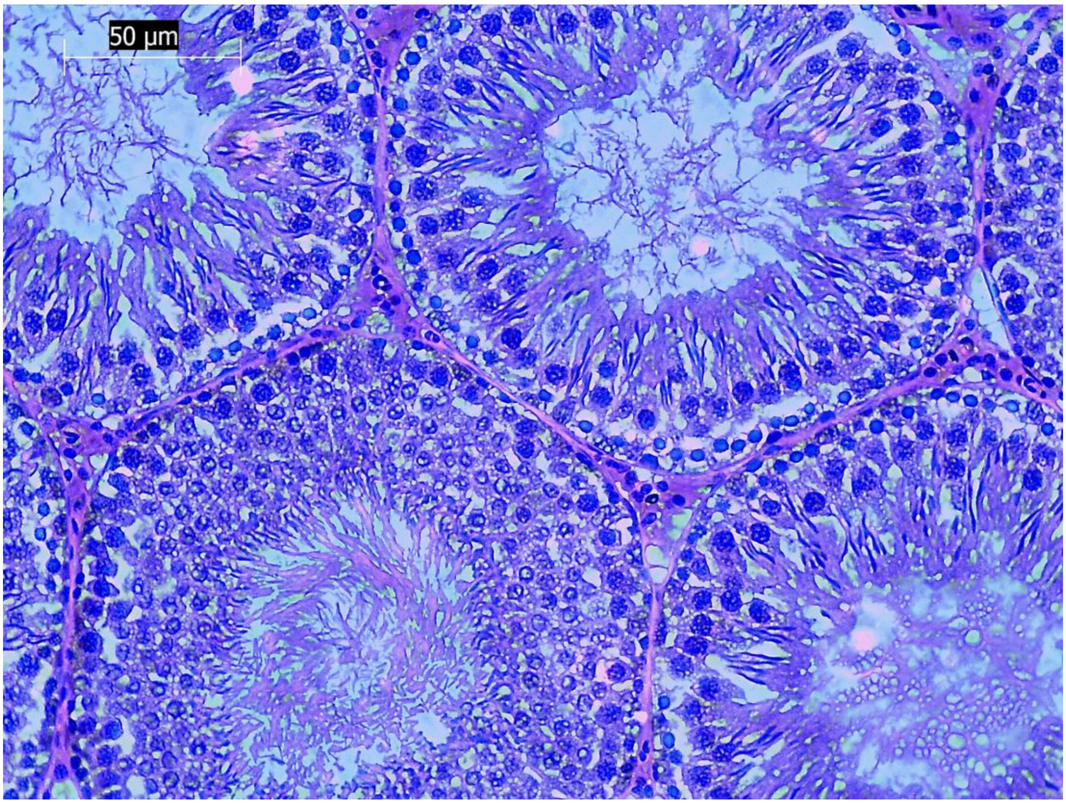

Figure S4. Original photomicrograph of Figure 2d.

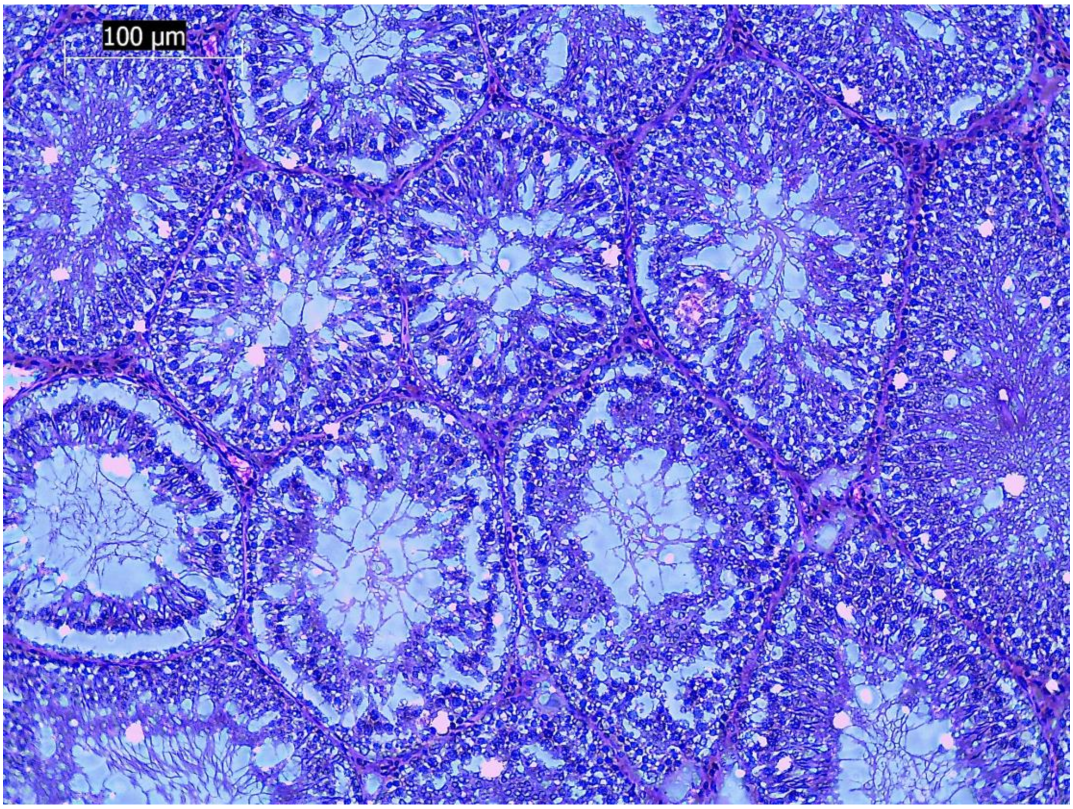

Figure S5. Original photomicrograph of Figure 2e.

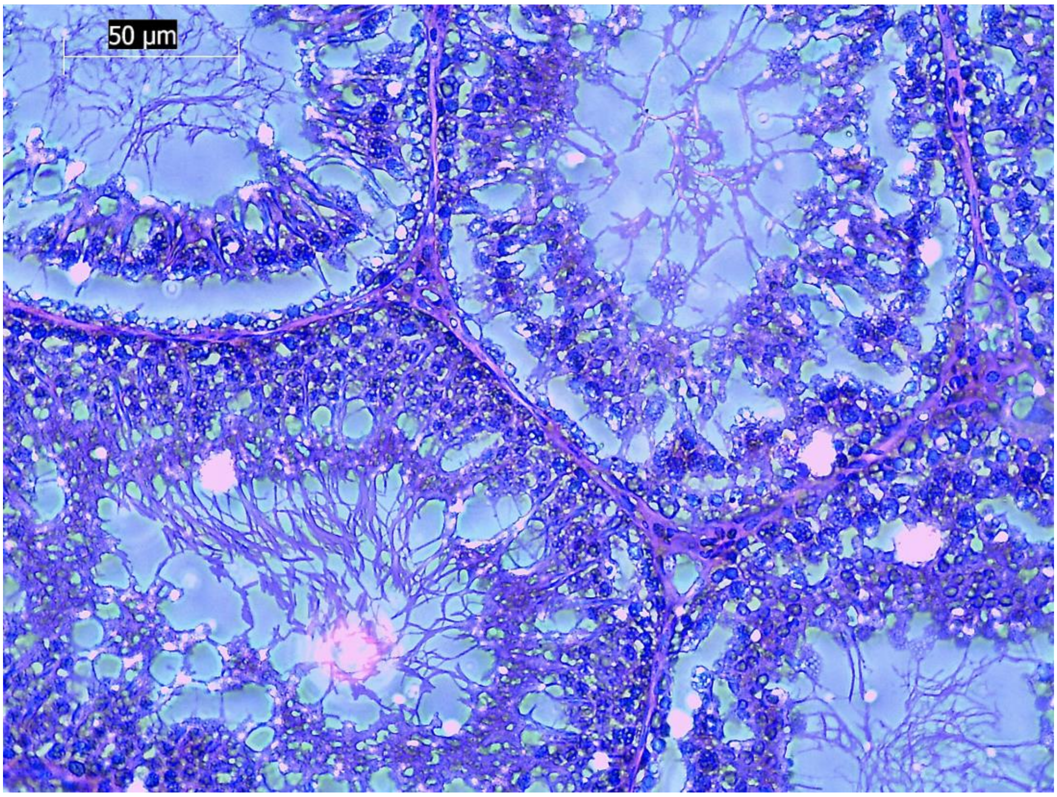

Figure S6. Original photomicrograph of Figure 2f.

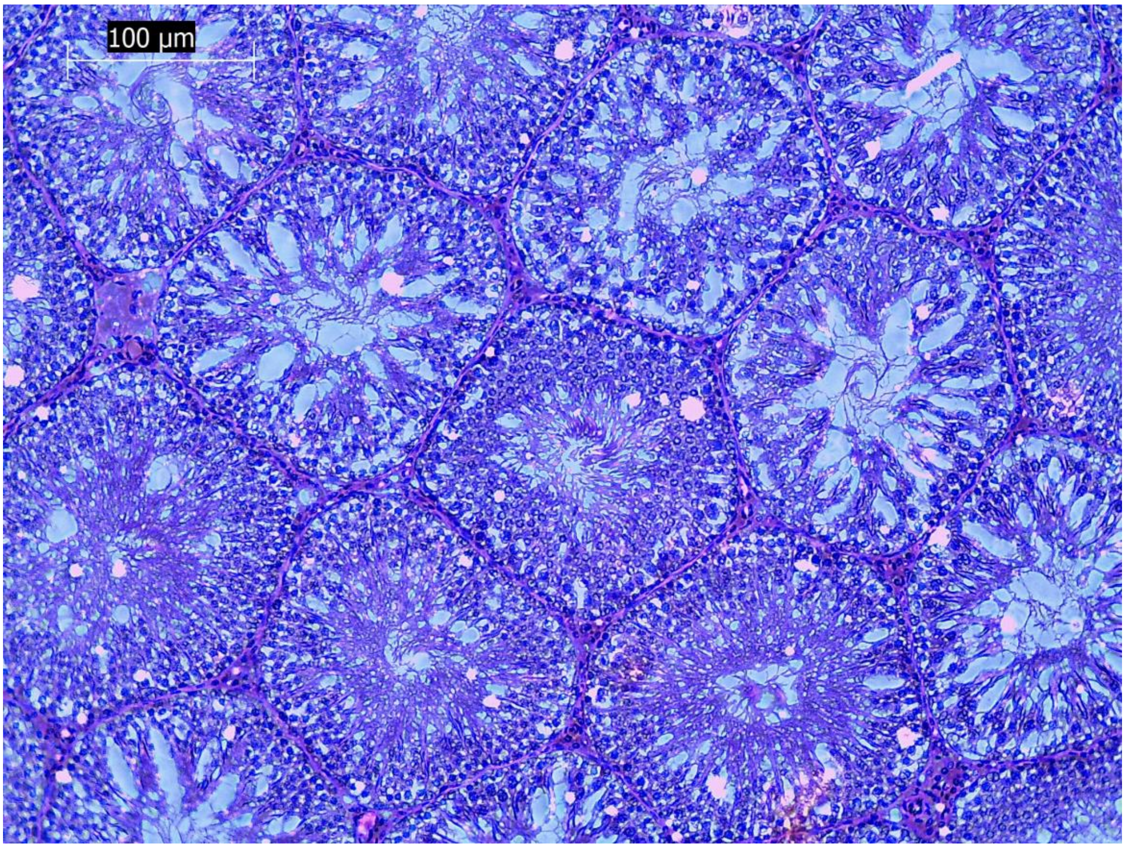

Figure S7. Original photomicrograph of Figure 2g.

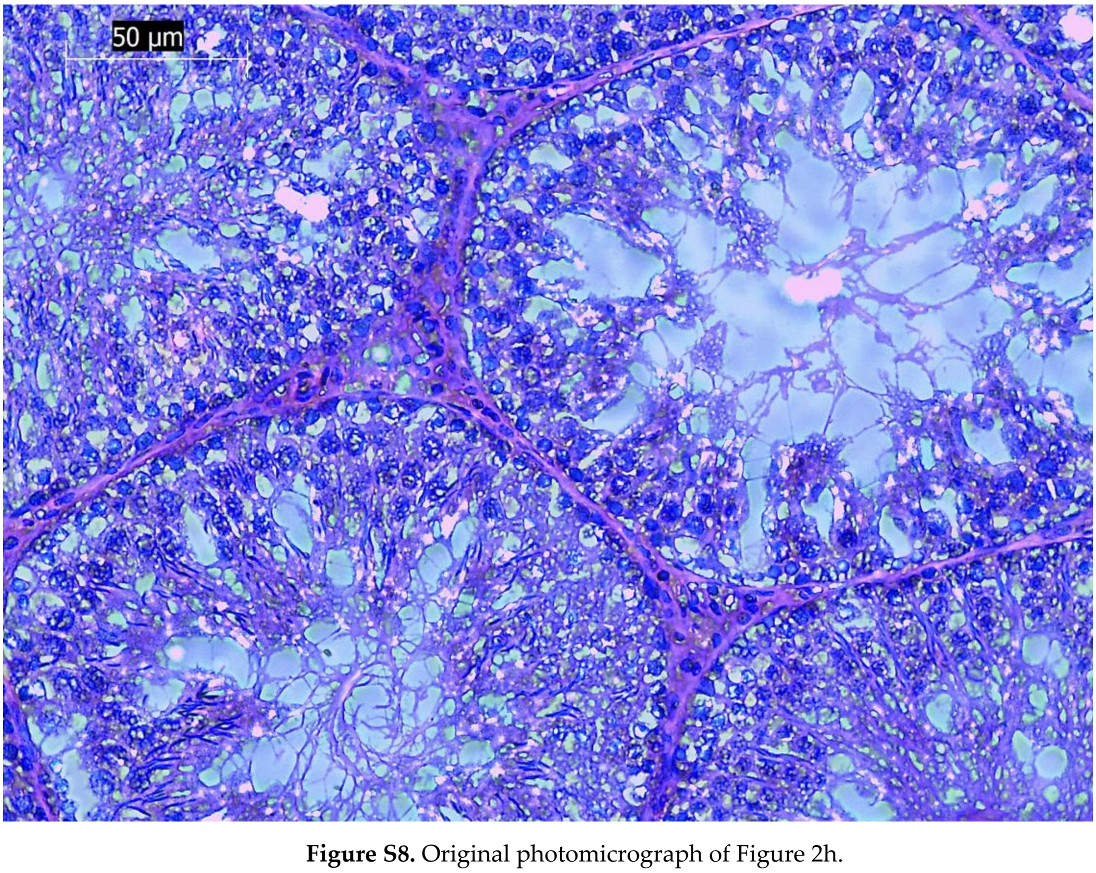

Figure S8. Original photomicrograph of Figure 2h.

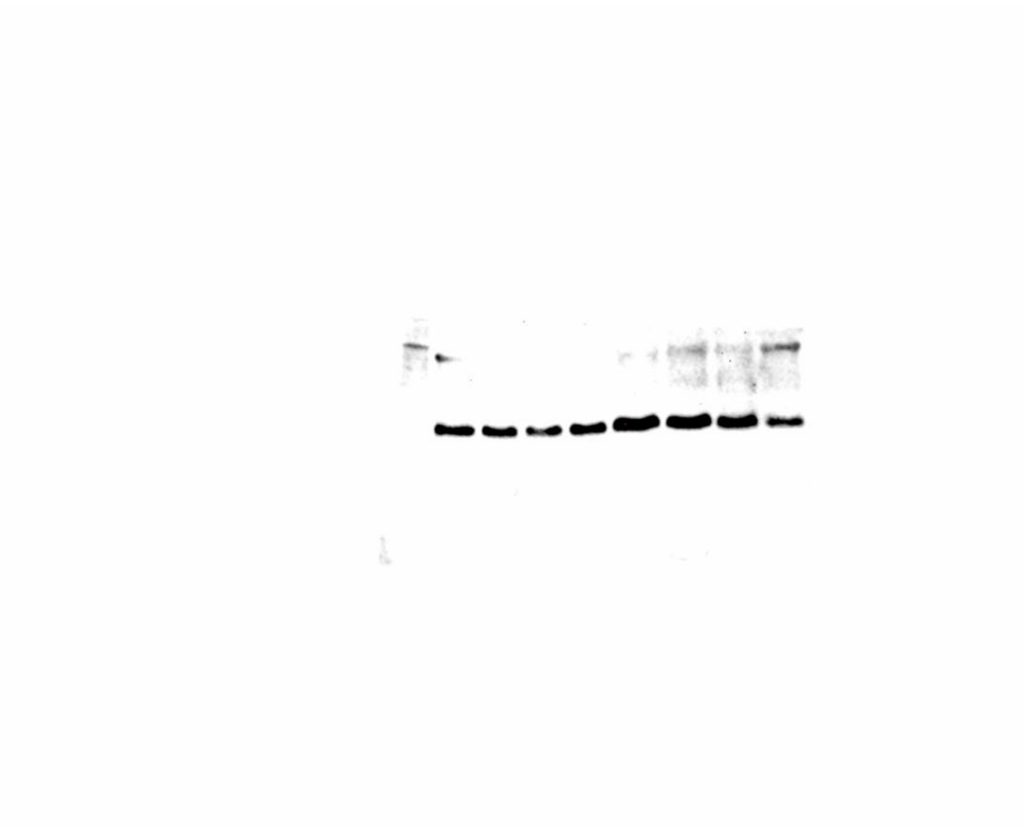

Figure S9. Original blot of BAX protein.

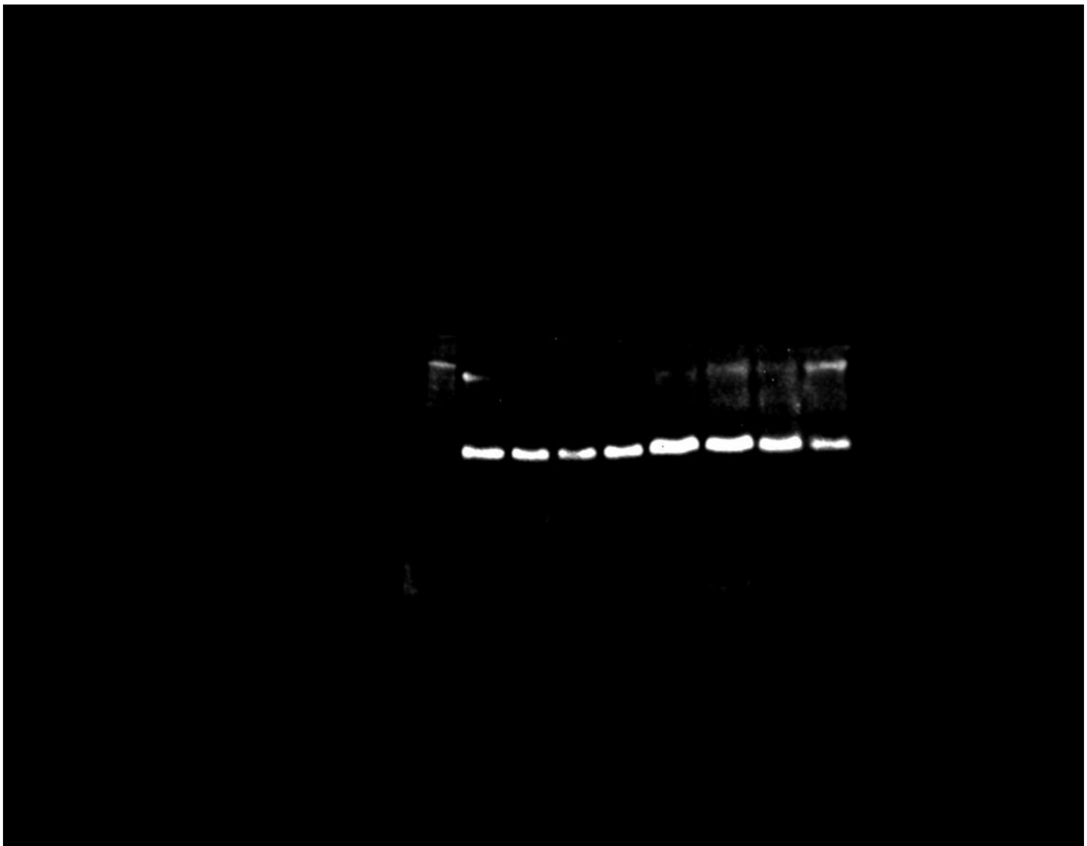

Figure S10. Inverted blot of BAX protein.

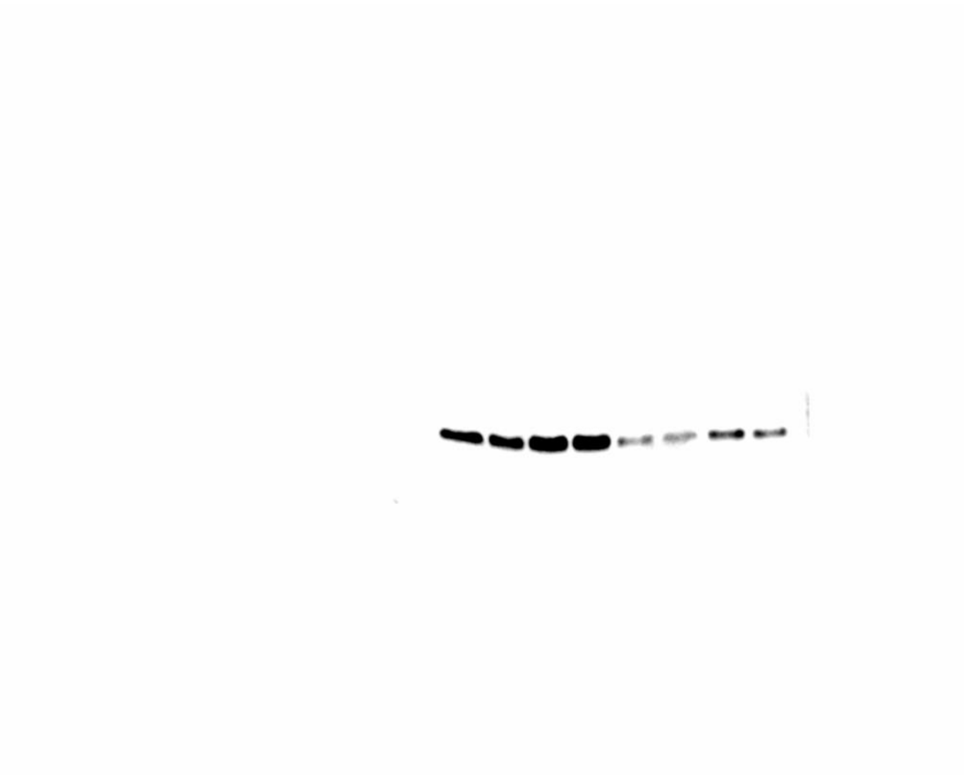

Figure S11. Original blot of Bcl-2 protein.

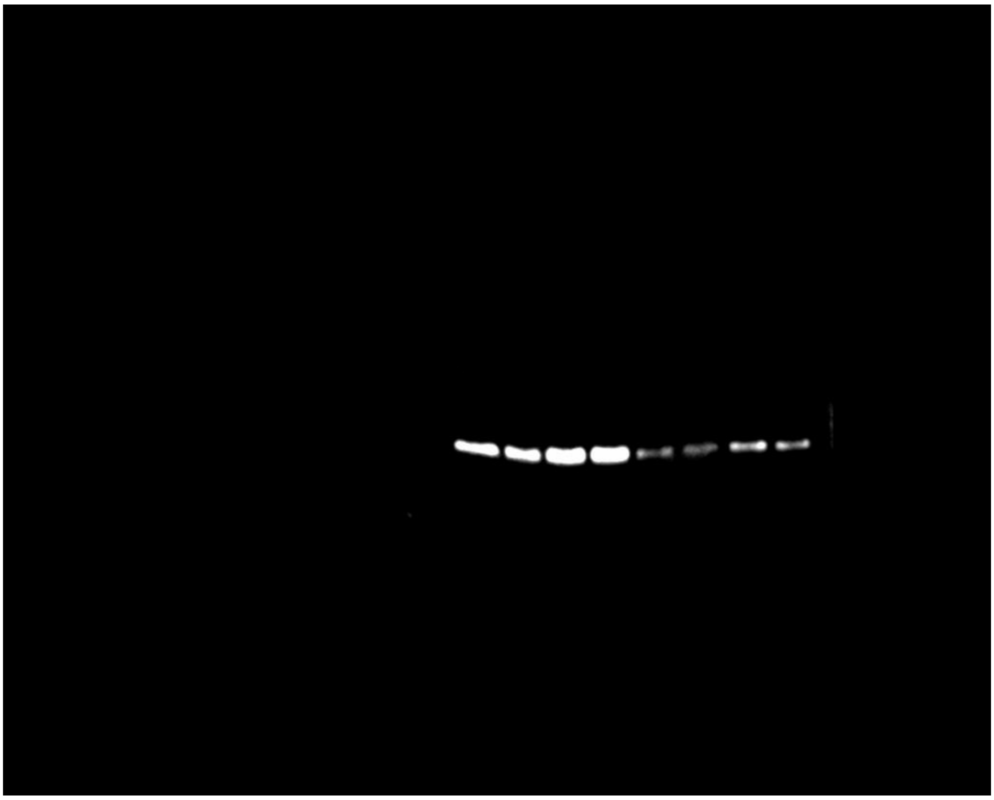

Figure S12. Inverted blot of Bcl-2 protein.

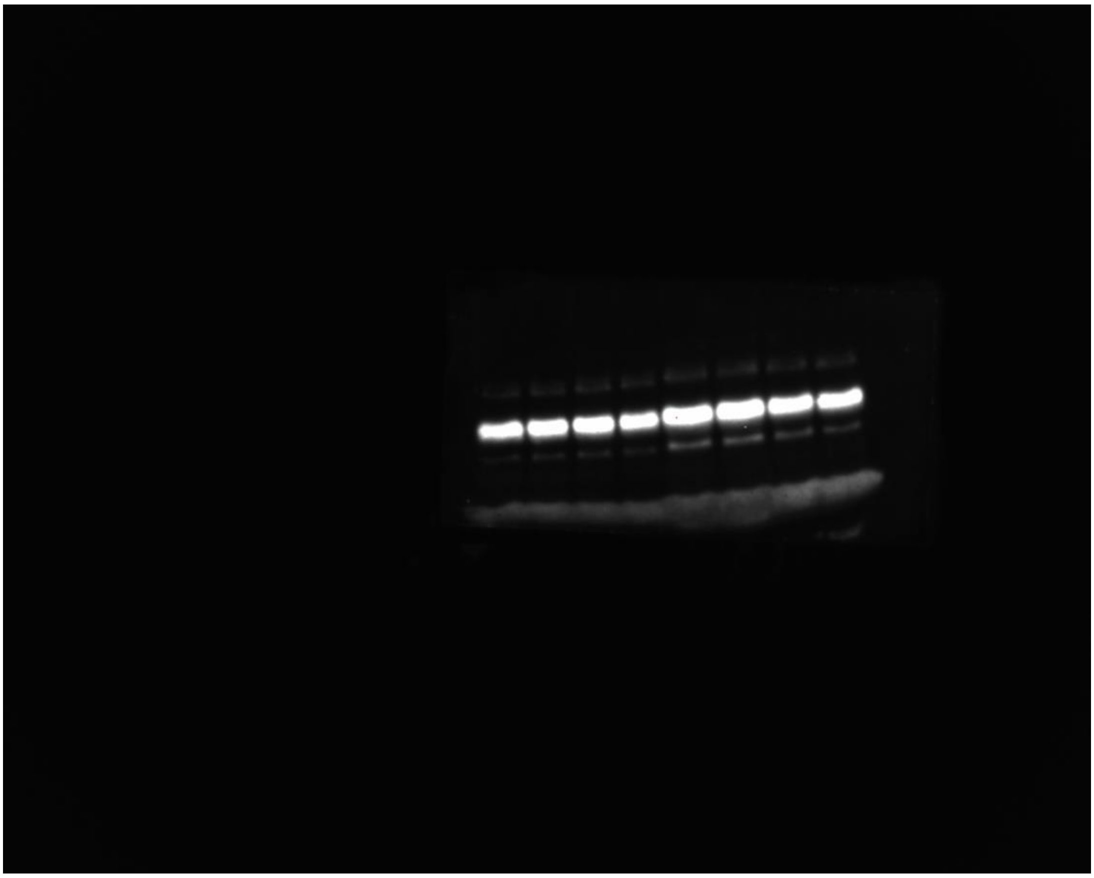

Figure S13. Original blot of p53 protein.

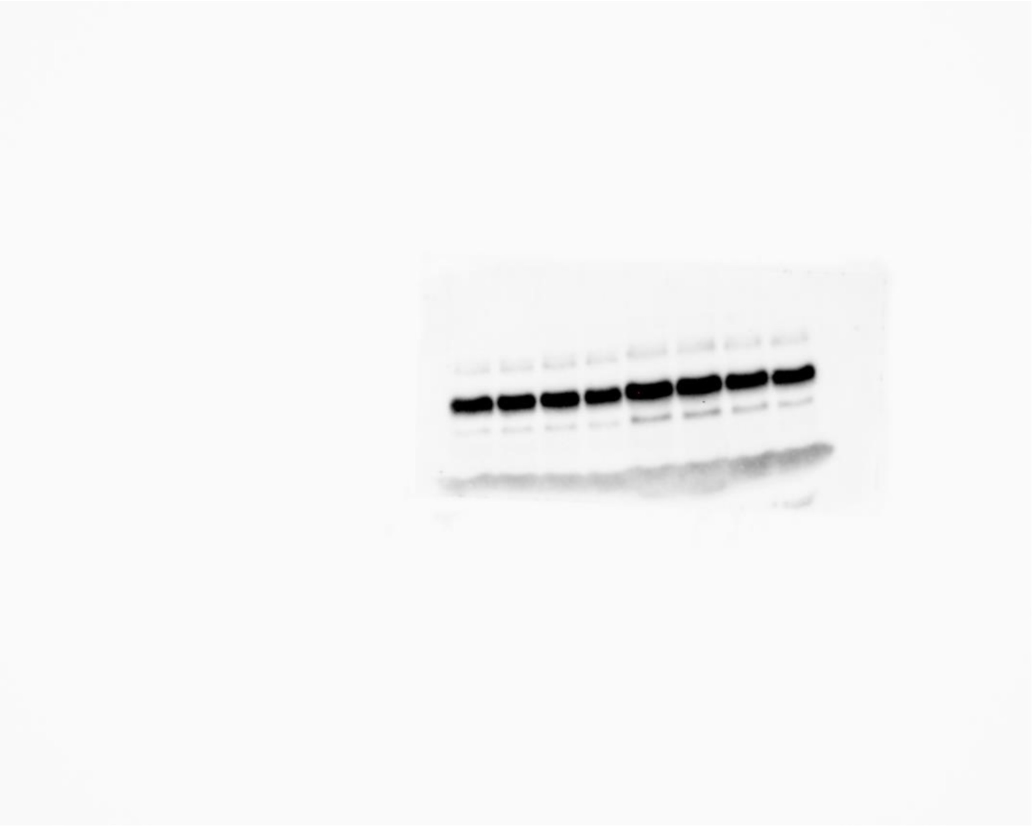

Figure S14. Inverted blot of p53 protein.

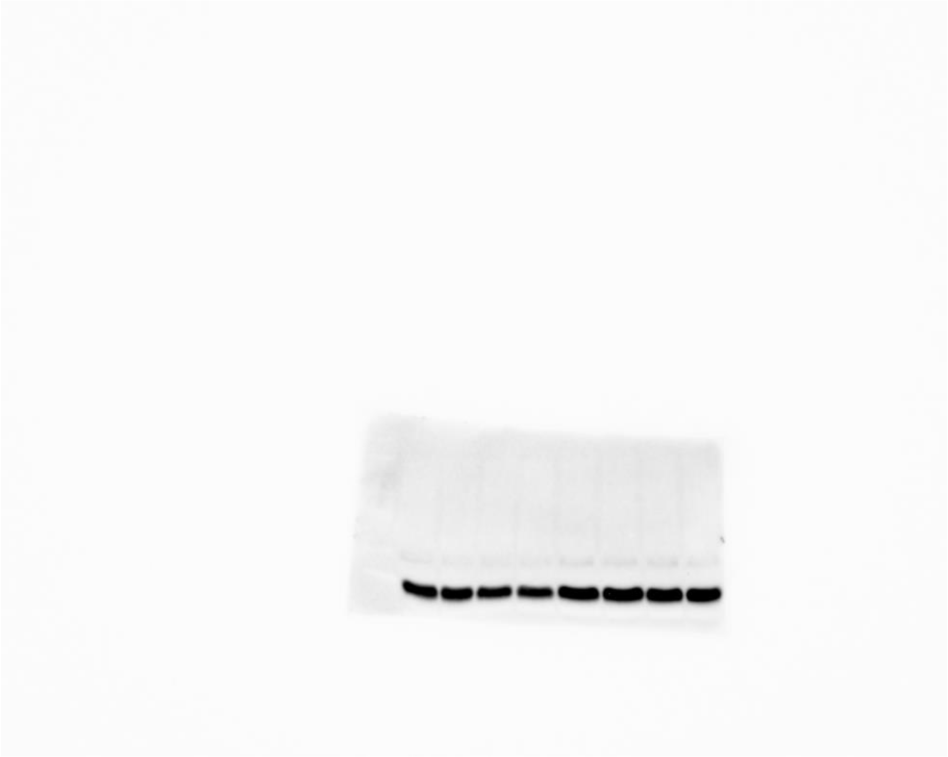

Figure S15. Original blot of caspase-3 protein.

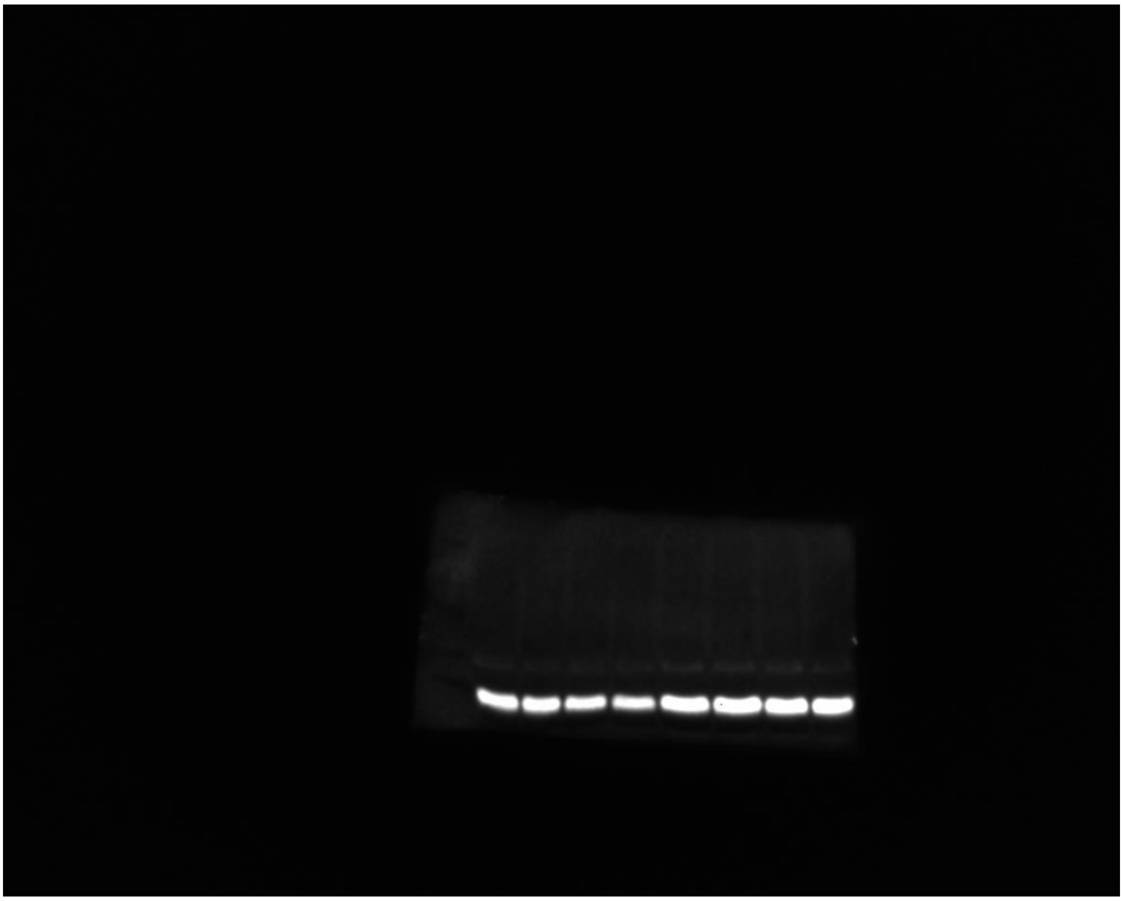

Figure S16. Inverted blot of caspase-3 protein.

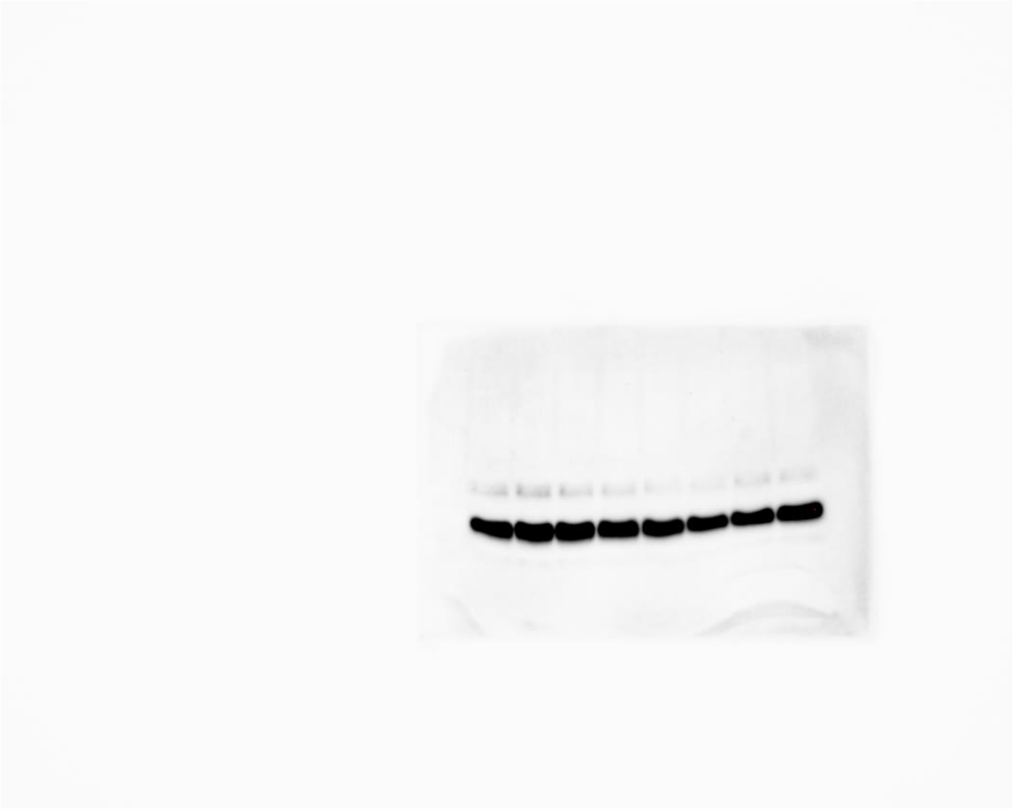

Figure S17. Original blot of beta actin.

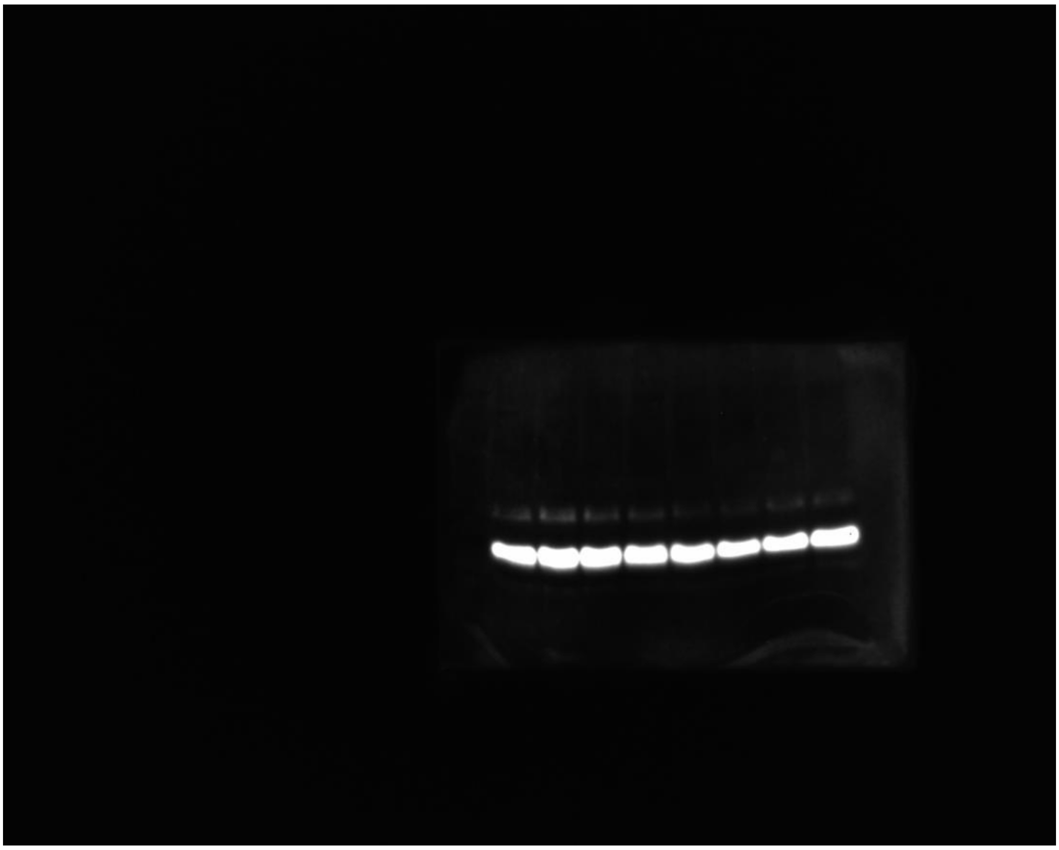

Figure S18. Inverted blot of beta actin.
